# Resting-Potential-Inspired Solid-State Iontronic Osmotic Power Source Enabled by Polarized MXene

**DOI:** 10.1007/s40820-026-02310-9

**Published:** 2026-07-22

**Authors:** Ziqi Ren, Long Zhang, Qixiang Zhang, Jianyu Yin, Mingfang Deng, Xubin Zhou, Qianqian Yao, Songzhan Li, Yihua Gao, Zhen Zhang, Nishuang Liu

**Affiliations:** 1 School of Physics and Wuhan National Laboratory for Optoelectronics (WNLO), Huazhong University of Science and Technology (HUST), Wuhan, 430074 People’s Republic of China; 2 Department of Applied Chemistry, School of Chemistry and Materials Science, University of Science and Technology of China (USTC), Hefei, 230026 People’s Republic of China; 3 State Key Laboratory of Bioinspired Interfacial Materials Science, Suzhou Institute for Advanced Research, University of Science and Technology of China (USTC), Suzhou, 215123 People’s Republic of China; 4 School of Electronic and Electrical Engineering, Hubei Collaborative Innovation Center of Textile Industrial Chain Generic Technology, Wuhan Textile University (WTU), Hubei, 430200 People’s Republic of China

**Keywords:** Osmotic energy, Resting potential, Polarized MXene, Solid-state

## Abstract

Bio-inspired osmotic energy has shown great potential as a portable energy source or artificial electrical organ for green and sustainable power generation. However, ion-electron decoupling problem and complex structures resulting from over-engineering often limit output power and portability. Here, we propose a comprehensive design strategy that exploits the unique ion-electron coupling properties of polarized MXene to mimic transmembrane ion transport at resting potential, thereby developing an all-MXene solid-state iontronics osmotic power source (IOPS). Density functional theory calculations and multiscale characterizations reveal that its operating mechanism is based on the diffusion dynamics of K^+^ under a concentration gradient and the Fermi level difference between two polarized MXene electrodes. The device achieves an initial open-circuit voltage exceeding 0.57 V and a high volumetric power density of 1030 μW cm^−3^. The highly integrated architecture of the IOPS module allows for straightforward scalability and reconfiguration to power commercial electronic devices. This work integrates the principles of biological ion gradients with emerging iontronics, providing a solid-state iontronic design strategy for compact osmotic power source. Here, taking the natural resting potential as a design blueprint, we report a solid-state iontronic osmotic energy generator, termed IOPS, which fully leverages the ion–electron coupling effect in polarized MXene. Its mechanism arises from the diffusion kinetics of K^+^ under an ion gradient and the Fermi level difference between two distinct polarized MXenes at the positive and negative electrodes. An initial open-circuit voltage exceeding 0.57 V and a high volumetric power density of 1030 μW cm^-3^ were achieved. This work integrates the fundamental principles of biological ion gradients with emerging iontronics technology, offering a paradigm for the practical deployment of osmotic energy.

**Supplementary Information:**

The online version contains supplementary material available at 10.1007/s40820-026-02310-9.

## Highlights

- The solid-state architecture of iontronics osmotic power source (IOPS) significantly enhances volumetric power density and eliminates risks associated with leakage and drying up.
- By making deep use of the ion-electron coupling properties of MXene, the highly integrated electrode design enables the IOPS device to be assembled into a compact osmotic power source using only three building blocks.
- IOPS arrays can efficiently power commercial electronic devices and show great potential in portable, responsive power systems.

**Supplementary Information:**

The online version contains supplementary material available at 10.1007/s40820-026-02310-9.

## Graphical abstract

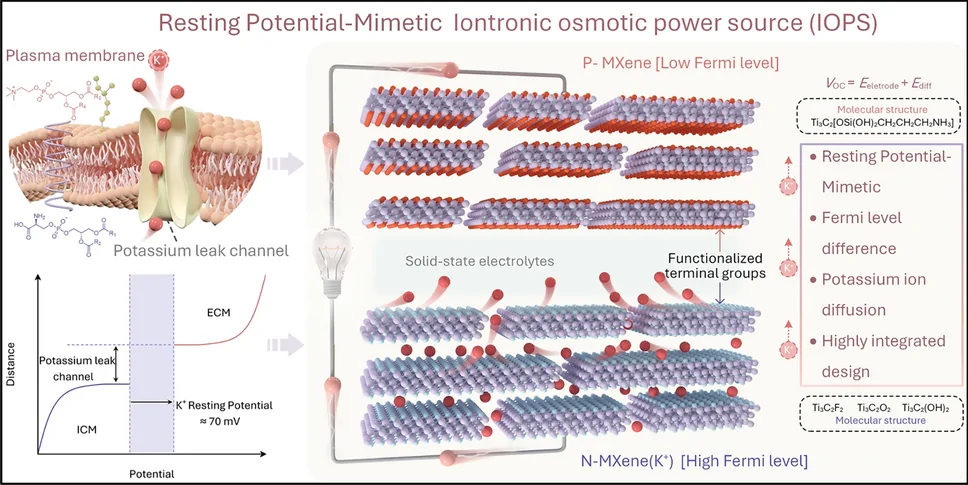

## Introduction

In nature, an "ionic language" mediates key processes such as signal transmission and energy modulation [1, 2]. Inspired by this biological intelligence, iontronics replicates the directional migration and dynamic control of ions from biological systems to achieve information processing, energy conversion, and device regulation in biomimetic devices [3, 4]. By integrating ion transport with electron conduction, iontronics has shown diverse and promising applications, from sensors to next-generation energy storage systems such as ion transistors, nanofluidic sensors, and osmotic energy [5–8]. Notably, osmotic energy is an emerging clean energy source, distinct from insertion batteries and supercapacitors [9–16]. It arises from entropy-driven directional ion migration, which releases Gibbs free energy that can be harnessed and converted into electricity. Bio-inspired osmotic energy has shown great potential as a portable energy source or artificial electrical organ for green and sustainable power generation [17–19]. The core of this biomimetic energy conversion mechanism lies in the precise control of the selective ion transport process, which is fundamentally similar to the principle of transmembrane potential generation in bioelectric organs.

**Fig. 1:**
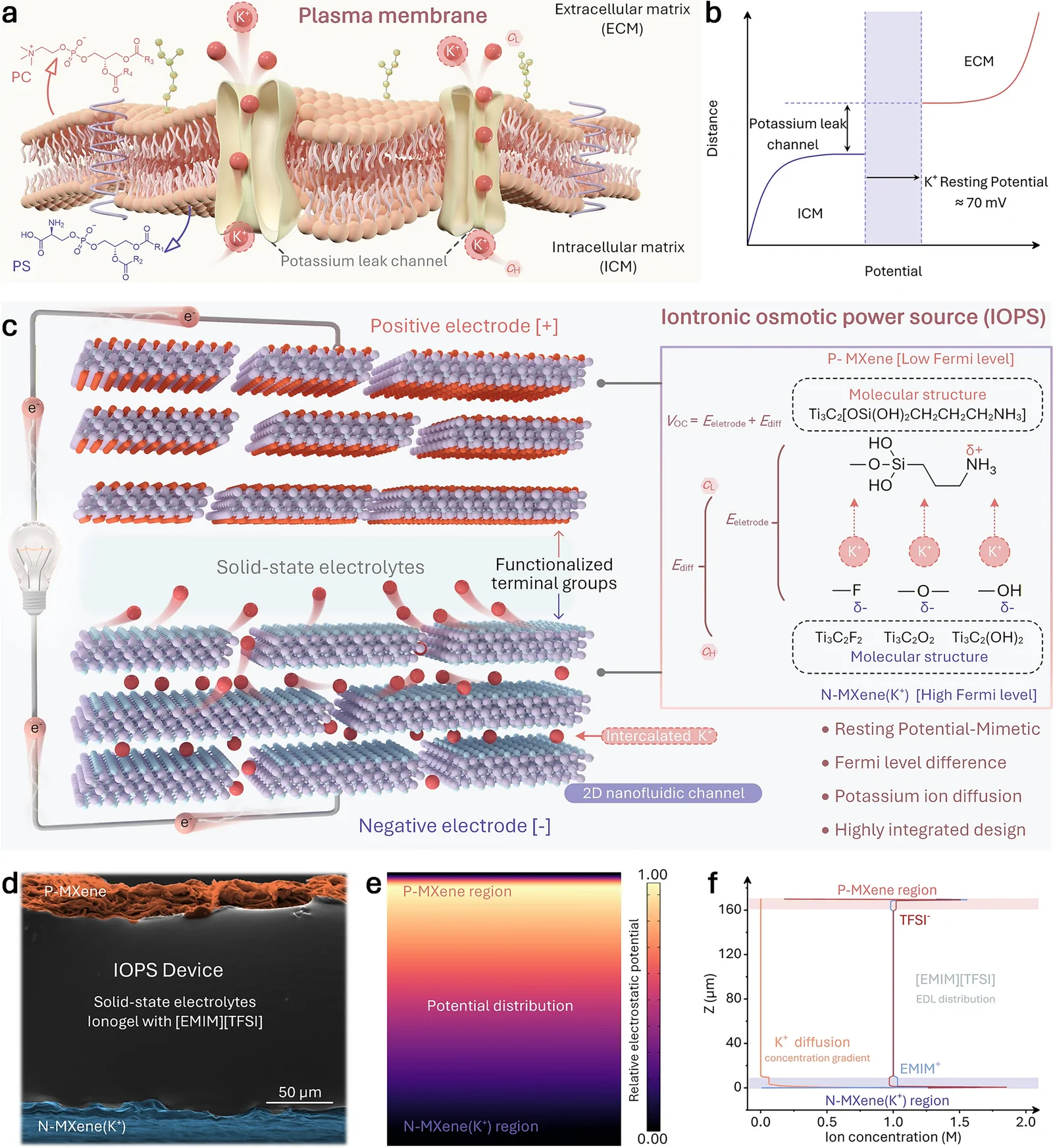
Design inspiration and working principle of solid-state IOPS. **a** K^+^ diffuses from ICM to ECM along the concentration gradient through potassium leak channels. **b** Formation of resting potential mainly depends on the following factors: the difference in ion concentration inside and outside the cell (mainly K^+^); the different permeability of potassium leak channels to different ions (K^+^ is the highest). **c** Schematic diagram of IOPS power generation. **d** SEM cross-sectional image of the IOPS device (orange and blue areas are P-MXene and N-MXene(K^+^) respectively). **e** IOPS potential distribution from finite element simulation. **f** Ion concentration distributions of K^+^, EMIM^+^, and TFSI.^–^ along the IOPS normal (z-axis)

Driven by this, researchers have explored a variety of bio-inspired membrane-based osmotic energy conversion systems and miniaturized osmotic energy conversion storage strategies [12, 20, 21]. For the latter, typical advanced work includes hydrogel-based soft power sources inspired by electric eels and energy sources utilizing the ion gradients between GO/rGO [17, 22]. However, current osmotic power systems based on iontronics often suffer from limited output power and poor portability. These limitations stem from two key factors: (1) the lack of intrinsic thermodynamic driving forces for efficient ion-electron coupling at the electrode–electrolyte interface, and (2) over-engineered device architectures that introduce unnecessary bulk and complexity. In contrast, natural electricity-generating systems have evolved optimal energy conversion modes through the spatial arrangement of ion channels and regulation of their local charge environments. Taking the formation of resting potential as an example, its core mechanism depends on potassium leakage channels on the cell membrane [23–26]. These channels establish a transmembrane potential difference by promoting the diffusion of K^+^ from the inside to the outside of the cell along the concentration gradient (Fig. 1a). In addition, the charge microenvironment of phospholipid molecules on the membrane surface can further regulate the polarity and amplitude of the resting potential (Fig. 1b) [27, 28]. This phenomenon provides important insights for the interface optimization of biomimetic ionic electronic devices.

Here, inspired by this natural mechanism, we exploit the unique ion–electron coupling properties of polarized MXene to mimic transmembrane ion transport at resting potential, thereby developing an all-MXene solid-state ion–electron osmotic power source (IOPS). The two-dimensional nanofluidic channels in pristine MXene provide nanoconfinement and co-ion shielding, establishing a key foundation for an “ionic language” system that mimics the biological resting potential generated by concentration gradients [29]. More importantly, MXene exhibits excellent electrical conductivity, typically exceeding 1000 S m^−1^ for electron conduction, and possesses a rich array of functional terminal groups (–OH,–F, and–O) for ion conduction [30, 31]. These unique properties enable MXene to simultaneously support ion storage, ion gradient transport, and electron transport. As shown in Fig. 1c, leveraging these unique advantages, we constructed the IOPS negative electrode (N-MXene (K^+^)) from Ti_3_C_2_T_x_ MXene pre-loaded with K^+^. Meanwhile, we leveraged the chemical tunability of MXene terminal groups to construct the IOPS positive electrode (P-MXene), ensuring a substantial Fermi level difference relative to the negative electrode. The solid-state electrolyte employs a cellulose-based ionogel that contains no water or organic solvents. This approach eliminates the risk of solvent evaporation and leakage in IOPS. Ultimately, the highly integrated electrode design enables the IOPS device to be assembled into a compact osmotic energy generator using only three building blocks. The essential reason is that we pre-install the ion storage chamber, which should be an independent module, directly into the electrode material, so the assembly complexity and part count are significantly lower than in comparable studies [12, 17, 18, 22, 32]. Figure 1d provides a cross-sectional scanning electron microscopy (SEM) image of the IOPS device. Density functional theory (DFT) calculations and multiscale characterizations reveal that alkali metal ions in the IOPS negative electrode form a distinct solvation structure and exhibit notable diffusion dynamics at the electrode–electrolyte interface. Meanwhile, the substantial energetic asymmetry between the two polarized MXene electrodes is consistent with asymmetric interfacial charge redistribution and EDL-like polarization at the electrode–ionogel interfaces, which contributes to the built-in interfacial potential. This interfacial potential synergizes with the ion diffusion gradient to yield a high initial open-circuit voltage (*V*_OC_) exceeding 0.57 V. This design enables the IOPS to achieve a maximum areal power density of 17.5 μW cm^−2^, an ultra-high volumetric power density of 1030 μW cm^−3^, and stable performance over 50 charge–discharge cycles (Table S1). In addition, finite element modeling of coupled Nernst–Planck and Poisson equations (Fig. S1, Table S2) quantitatively confirms the IOPS power generation. Figure 1e provides a visualization of the potential distribution in the IOPS (Note S1.1). Dynamic simulation results of 4 ps are used to analyze the diffusion and transport behavior of ions within the IOPS (Fig. 1f), further supporting the proposed mechanism. Thanks to the customizable nature of IOPS, scalable integration of its units can be readily achieved. IOPS arrays can effectively power commercial electronic devices and demonstrate significant potential in portable, responsive power systems. This work is expected to provide a new paradigm for energy storage in ionic electronic devices.

## Experimental Section

### Materials

Lithium fluoride (LiF), 3-aminopropyltriethoxysilane (APTES), and ionic liquid [EMIM][TFSI] were purchased from Aladdin. Ethanol (C_2_H_5_OH), hydrochloric acid (HCl), potassium hydroxide (KOH), sodium hydroxide (NaOH), lithium hydroxide (LiOH), and potassium chloride (KCl) were purchased from Sinopharm Chemical Reagent Co., Ltd. The cellulose membrane was purchased from Jinteng Co., Ltd. Ti_3_AlC_2_ MAX phase (600 mesh) was purchased from 11 Technology Co., Ltd. Deionized water (resistivity > 18.2 MΩ cm) was collected from an ultrapure water system.

### Preparation of N-MXene Membrane

First, 4.00 g of LiF powder was gradually added to 80.0 mL of HCl solution (9.00 M) under an ice bath. Then 6.40 g of Ti_3_AlC_2_ MAX phase was slowly added to the above mixed solution in batches. The reaction was continued at 35.0 °C for 24.0h to etch the Al atomic layer. After the reaction, centrifuge and wash repeatedly with deionized water until pH > 6.00, at which time the supernatant was dark green. Finally, ultrasonic treatment was performed in an ice bath under N_2_ atmosphere for 40.0 min, and centrifugation was performed at 3000 rpm for 10.0 min to obtain a MXene solution containing single-layer nanosheets with a concentration of 10.0 mg mL^−1^. The obtained MXene solution was vacuum filtered and dried to obtain an N-MXene membrane, and its weight was recorded as *m*_Pristine_.

### Preparation of N-MXene(K^+^), N-MXene(Na^+^) and N-MXene(Li^+^) Membranes

The obtained N-MXene membranes were immersed in 0.2 M KOH, NaOH or LiOH solutions for different periods of time and sealed. After waiting for the alkali metal ions to be inserted into the MXene interlayer, the membrane was taken out and washed by soaking in ultrapure water, and then the excess solution was removed on a spin coater. After drying, the membrane was weighed. The weight percentage of the inserted alkali metal ions can be calculated approximately as: (*m*_meta_–*m*_Pristine_)/*m*_Pristine_.

### Preparation of P-MXene Membrane

The obtained MXene solution was completely dehydrated by high-speed centrifugation at 10,000 rpm and redispersed in ethanol to prepare a suspension. Acetic acid was then added to reduce the pH to below 4 and a small amount of ultrapure water was added. APTES was then slowly added dropwise and reacted for 24h. The mass ratio of the additives was MXene:H_2_O:APTES:ethanol = 1:2:2:100. After the reaction was completed, the product was washed with ethanol and water respectively. The P-MXene finally obtained was redispersed in water, and a P-MXene membrane was obtained by vacuum filtration and drying.

### Preparation of Adhesive Ionogel

The commercially available cellulose filter was soaked in the ionic liquid [EMIM][TFSI] for 3 min, and then spun at 500 rpm for 5s and 5000 rpm for 30s by a spin coater to remove the excess ionic liquid. This step is intended to remove unbound liquid from the surface, not to eliminate the ionic liquid retained within the cellulose ionogel. The retained [EMIM][TFSI] is a necessary component of the solid electrolyte, providing the ion-transport medium, whereas excessive free liquid could cause leakage, variable interfacial contact, and unstable output. The cellulose membrane can be quickly prepared into an adhesive ionogel within 1h. The weight percentage of the ionic liquid is calculated as follows: (*m*_Cellulose@IL_–*m*_Cellulose_)/*m*_Cellulose_. The average weight percentage of the ionic liquid in the prepared adhesive ionogel is about 290%. According to the density of [EMIM][TFSI] (1.53 g cm^−3^), each square centimeter of cellulose filter can load about 10 μL of ionic liquid. Other ionic liquids such as [EMIM][Ac] and [EMIM][OTf] have also been used, however they completely dissolve the cellulose membranes because they completely restructure the inter-cellulose hydrogen bonds.

### Assembly of IOPS

For basic power generation performance testing, the MXene-based membrane was cut into 1.5 cm × 1 cm size and pasted on a single-sided release PI film. Subsequently, the pre-prepared adhesive ionogel was cut into 1 cm × 1 cm size. Finally, different combinations of MXene-based membrane were pasted on both sides of the ionogel to form an IOPS unit with an effective test area of 1 cm^2^. It is worth noting that ion diffusion may occur immediately after the device is assembled, so relevant research needs to be carried out immediately after the device is assembled. Other IOPS assembly methods are carried out according to actual application requirements.

### Electrical Measurements

Power generation measurements were performed under ambient conditions using a Keithley 2450 source meter (Keithley Instruments, Inc., USA). The current of the circuit parameters for the voltage output test was set to 0 nA, and the voltage of the circuit parameters for the current output test was 0 V. Constant voltage charge/discharge curves were tested using Keithley 2450 with a charge voltage of 0.5 V and a discharge voltage of 0. Electrochemical tests were performed on a CHI 660D electrochemical workstation (CH Instruments, Inc.). The electrochemical impedance spectroscopy of IOPS was performed in the frequency range of 0.01–100 kHz with an AC amplitude of 5 mV.

### Characterizations

Transmission electron microscopy (TEM, FEI Titan G260-300), SEM (FEI Nova Nano-SEM450), atomic force microscopy (AFM, SPM 9700) were used to analyze the surface morphology and chemical composition. Kelvin probe force microscopy (KPFM, Bruker Dimension Icon) was used for CPD test. X-ray diffraction (XRD, 08 x’pert3 powder), X-ray photoelectron spectroscopy (XPS, Thermo ESCALAB 250XI), Fourier transform infrared spectroscopy (FT-IR, Thermo Fisher Scientific Nicolet iS20) and zeta potential analyzer (DLS Malvern Zetasizer Nano ZS90) were used to further characterize the composition of the compounds.

### Calculation Method

All DFT calculations were performed using the generalized gradient approximation (GGA) of the Perdew–Burke–Ernzerhof (PBE) function. The convergence criteria for the ideal optimized structure were set to Fine, with k-points set to 3 × 3 × 1, energy cutoff set to 500 eV, and convergence criteria for the electronic self-consistent loop and force set to 0.01 meV and 0.01 eV Å^−1^, respectively. The 3 × 3 supercell structures of four MXenes with different terminal groups were constructed, with the K^+^ terminal occupying the *fcc* site. The binding energy is calculated as follows:

$$\begin{array}{*{20}c} {E_{{\mathrm{b}}} = E_{{{\mathrm{MXene}} - {\mathrm{K}}}} - \left( {E_{{{\mathrm{MXene}}}} + { }E_{{\mathrm{K}}} } \right)} \\ \end{array}$$

Here, Eb represents the binding energy, EMXene-K is the total energy of potassium ions adsorbed on the MXene surface, EMXene is the energy of pure MXene, and EK is the energy of a single potassium ion.

Ionogel and K^+^ interaction structures were constructed. K^+^ diffusion trajectories from the MXene surface to the ionic liquid were constructed (4 ps NVT runs were performed at 298 K). Structural optimization was performed first, followed by binding energy calculations.

## Results and Discussion

### Design Principles and Manufacturing of Solid-State IOPS

**Fig. 2:**
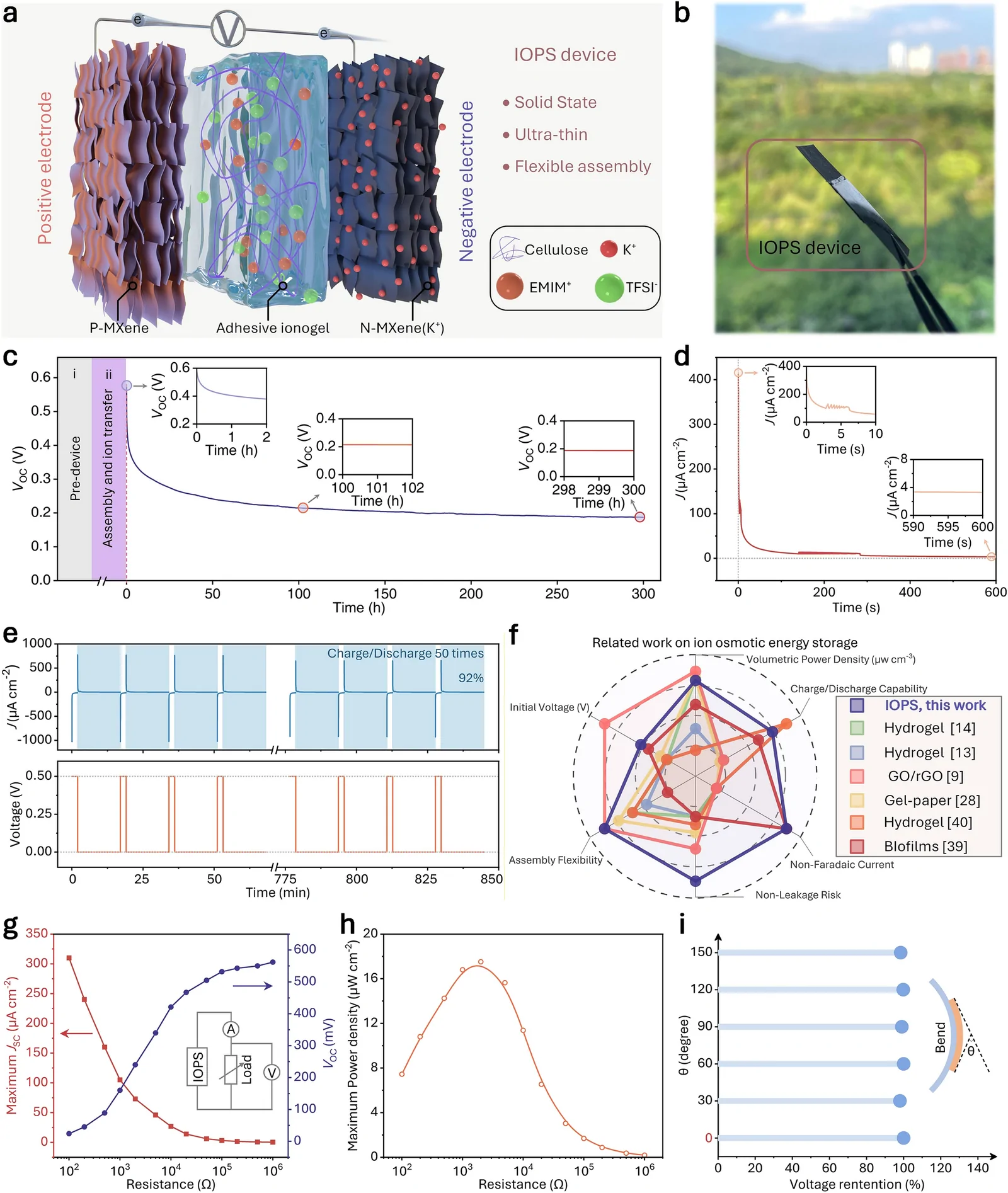
Electric output performance of a IOPS unit. **a** Assembly and testing scheme of IOPS. **b** Optical photo of the IOPS device under natural light. **c** Open-circuit voltage (*V*_OC_) of IOPS device. **d** Output short-circuit current (*J*_sc_) after assembling the IOPS device. **e** Cyclic current and time records of IOPS device short-circuited and discharged 50 times after 0.5 V constant voltage charging. **f** Multidimensional data visualization of ion osmotic energy storage from related work. **g** Voltage and area current density output of an IOPS with a variable resistor. **h** Maximum power density output of IOPS with variable resistor. **i** Voltage retention rate of IOPS at different bending angles (based on the initial voltage in the previous text, the same below)

The solid-state IOPS device was constructed by two polarized MXene electrodes and a cellulose-based ion gel (Fig. 2a, b). Atomic force microscopy (AFM, Fig. S2) revealed the microstructure of the pristine MXene nanosheets, which were approximately 1.12 nm thick and 1.0 μm in size. The functionalized terminal groups (–F, –O,  –OH) of the pristine MXene possess high electronegativity, inducing a surface partial negative character (*δ* −). Therefore, we designate this electrode as Negative Polarized MXene (N-MXene) [30, 33]. Free-standing membranes can be prepared by vacuum-assisted filtration, and the two-dimensional nanosheet confined channels in the membrane can serve as a natural warehouse for the storage of alkali metal ions. The N-MXene(K^+^) membrane was fabricated by sealing and immersing the N-MXene free-standing membrane in a high-concentration KOH solution (Note S1.2) [20, 34, 35]. The control experiment (Fig. S3) shows that when the immersion time exceeds 48h and the immersion concentration exceeds 0.20 M, the storage capacity of K^+^ no longer increases, and its maximum storage capacity is about 5.2 wt% (relative to N-MXene membrane). As mentioned above, N-MXene(K^+^) serves multiple roles in solid-state IOPS, including as a cation-selective permeable membrane, ion reservoir, current collector (high conductivity), and negative electrode.

Currently, the most widely used synthesis method of Ti_3_C_2_T_x_ MXene is MILD method or direct HF etching, which easily generates negative charges from surface terminals. Consequently, reports on Positive Polarized MXene (P-MXene) featuring positive surface polarity are relatively scarce. In our work, P-MXene was prepared by using the silane coupling agent APTES to form O–Si covalent bonds with the terminal hydroxyl groups of MXene under acidic conditions (Note S1.2) [36, 37]. Since APTES contains three ethoxy groups, its products include five forms: monodentate, bidentate, tridentate, lateral polymerization and hydrogen bonding (Fig. S4). The preparation methods of N-MXene(K^+^) and P-MXene are detailed in Fig. S5 and the Methods section. The microscopic morphologies of N-MXene and P-MXene were revealed by transmission electron microscopy (TEM) and selected area electron diffraction (SAED) (Fig. S6). Ion transport properties across N-MXene and P-MXene membranes were assessed using a dual-chamber electrochemical cell with Ag/AgCl electrodes, capturing *I*–*V* curves under a 0.50/0.010 M KCl concentration gradient (Fig. S7). Calculations gave anion and cation migration numbers for P-MXene and N-MXene as *t*_Cl_ = 0.750 and *t*_K_ = 0.976, respectively (Note S1.3), confirming the transport and storage properties of anions and cations in the two MXene membranes. Figure S8a shows the optical images of the three MXene membranes under natural light. Scanning electron microscopy (SEM) images and elemental mapping by energy dispersive spectroscopy (EDS) show the microstructure and uniform distribution of the main atoms on the surface of the P-MXene and N-MXene (K^+^) membranes, respectively (Fig. S8b). The zeta potential of N-MXene, N-MXene(K^+^), and P-MXene solutions (0.1 mg mL^−1^), as shown in Fig. S8c, reflects the difference in their surface potentials. The Zeta potential results reflect the altered electrokinetic surface environment after KOH treatment and APTES modification. The 2θ angle shift of the X-ray diffraction (XRD) pattern (Fig. S9) reveals the intra-membrane spatial differences of N-MXene, N-MXene(K^+^), and P-MXene (Note S1.4). X-ray photoelectron spectroscopy (XPS) results, shown in Fig. S10 and Table S3, confirm the presence of typical elements: K in N-MXene(K^+^) and Si and N in P-MXene, indicating successful synthesis (Note S1.5). Characteristic absorption peaks from Fourier transform infrared spectroscopy (FT-IR, Fig. S11) also corroborate these findings (Note S1.6). Sheet resistivity of N-MXene, N-MXene(K^+^), and P-MXene films was measured using the four-point probe technique (Table S4). Despite undergoing intercalation and chemical modification, the membranes maintained excellent metallic conductivity.

For the fabrication of solid polymer electrolytes (SPE), the good thermal stability, low volatility, non-flammability and high ionic conductivity of ionic liquids are fully utilized [38, 39]. Here, we developed a fast method to fabricate adhesive ionogel through random copolymer networks formed by ionic liquids [EMIM][TFSI] and cellulose membranes [40]. As shown in Fig. S12, [EMIM][TFSI] was able to partially restructure the hydrogen bonds of cellulose polysaccharide units and gel the macroscopic cellulose membrane without using any organic solvents. This is a very simple, rapid and direct method for preparing SPE. It should be noted that the ionogel in this study was rapidly prepared using a commercially available cellulose membrane, and the actual membrane thickness determined the final thickness of the ionogel. Therefore, the selected thickness should be considered as a practical design choice based on the rapid preparation method via commercial cellulose membranes, rather than a universally applicable optimal value. The prepared cellulose-based ionogel is essentially a solid mixture in a liquid-like dissipative state. The optical image and SEM image of the adhesive ionogel formation process are shown in Fig. S13. The porous cellulose membrane can quickly form a uniform ion gel within 1h and maintain its original shape after two weeks. Figure S14 shows the conductivity-temperature dependence characteristics of the ion gel at test temperatures of -10 to 80 °C. When it is at room temperature 25 °C, the ionic conductivity can reach 1.07 mS cm^−1^. In addition, the ion gel has a strain tensile strength of about 160% and a strong adhesion force, and it is not easy to cause interfacial delamination between the electrode and the electrolyte, which ensures its stability when mechanical deformation occurs (Fig. S15). After being stored in ambient conditions for one month, the ion gel still maintains its shape and transparency and has no corrosive effect on metals (Fig. S15c).

### Electric Output Performance of a IOPS Unit

In all electrical measurements, MXene-based electrodes function effectively as both electrodes and current collectors without inducing any interference reactions, owing to their outstanding intrinsic conductivity. In a test environment set at approximately 25 °C and 60% humidity, the electrical output of a single generator, activated by wetting of ionogel interface infiltration, was measured, revealing a continuously decreasing open-circuit voltage (*V*_OC_) (Fig. 2c). Initially at 0.579 V, the device's voltage retention decreased to 69.6%, 59.5%, 41.3%, and 32.3% after 1, 10, 100, and 300h, respectively. This phenomenon occurs as the IOPS system spontaneously progresses toward thermodynamic equilibrium, where K^+^ migration persists at the anode interface even under open-circuit conditions, driving the *V*_OC_ to asymptotically approach the equilibrium potential through dynamic ionic concentration balancing within the device. This process is driven by the entropy increase effect, and the system tends to a high-entropy uniform distribution state [41]. However, when the test was carried out for 300h, we observed an interesting phenomenon. The *V*_OC_ of IOPS at this time remained at about 0.187 V and almost stopped decreasing, while similar ion gradient work usually dropped to zero. We believe that this abnormal phenomenon is because the diffusion of K^+^ tends to dynamic equilibrium, and the downward trend of *E*_diff_ is almost completely suppressed. At this quasi-steady stage, the diffusion-related contribution is largely suppressed, and the remaining *V*_OC_ is mainly maintained by the built-in interfacial potential arising from the heterogeneous Fermi levels of P-MXene and N-MXene(K^+^). This residual interfacial potential should be distinguished from the initial extractable diffusion-driven output, which can be recovered after re-establishing the non-equilibrium K⁺ distribution. After the K^+^ diffusion gradient is largely relaxed, IOPS enters a quasi-steady state in which a residual *V*_OC_ may still be maintained by the heterogeneous electrode pair, whereas the major diffusion-driven power contribution can be restored only after re-establishing the nonequilibrium ion distribution by external charging. Furthermore, we investigated the electrical output performance of other alkali metal ions. Using a similar alkalization-assisted intercalation method, Li and Na, the same main group elements of K element, were inserted into the MXene interlayer (Fig. S16). Notably, N-MXene(Li^+^) and N-MXene(Na^+^) exhibited higher initial voltages (0.621 and 0.601 V) and varied voltage decay rates, potentially due to differences in Fermi level energy and ion solvation sheath diffusion rates from distinct ion intercalations [42]. Figure 2d documents the energy dissipation of IOPS during short-circuit measurements. The initial peak short-circuit current density (*J*_SC_) reached 415 μA cm^−2^, as K^+^ is depleted in the negative electrode–electrolyte interface, rapidly decreasing with a half-life of 20s. After operating for 3600s, the *J*_SC_ remained above 3.29 μA cm^−2^. In addition, the current and voltage performance of IOPS has good cyclability. The IOPS underwent a 15-min discharge under short-circuit conditions followed by a 2-min charge at 0.5 V. Subsequently, the current signal was recorded for 50 cycles with an alternating bias of 0.5 | 0 V, and the IOPS recovered more than 92% of the original capacity during this discharge cycle (Figs. 2e and S17). This demonstrates that a 0.5 V charging step can restore the non-equilibrium K^+^ distribution and recover the diffusion potential contribution by driving K^+^ back to the N-MXene side. We have also visualized data from major recent works related to ion osmotic energy storage, which showed that IOPS is in a relatively leading position in several aspects (Fig. 2f) [12, 17, 18, 32, 43, 44].

Based on the maximum power transfer theorem (MPTT), the system achieves peak power output when load resistor (*R*_Load_) matches the system’s internal resistance. To evaluate the energy harvesting capabilities of IOPS, we measured the electrical energy generated by connecting an adjustable load resistor (*R*_Load_) to an external circuit (Fig. 2g). The peak power density was obtained from the measured *P* = *J* × *V* curve under different external loads. Figure 2h shows that for a 1 cm^2^ generator, the optimal resistor load is 2 kΩ, which achieves peak power densities of 17.5 μW cm^−2^ and 1030 μW cm^−3^ (Table S1). The volumetric power density was calculated from the maximum measured areal power density divided by the effective device thickness (the effective testing area was 1 cm^2^, and the total device thickness was approximately 170 μm based on the cross-sectional SEM image). Among these, the ultra-high volumetric power density of IOPS is particularly noteworthy, which is due to the ultra-thin thickness of the IOPS device. Additionally, IOPS exhibited a fairly high potential for flexible devices, with only slight performance fluctuations under bending deformation from 0° to 150°, which means it is resistant to mechanical deformation (Fig. 2i). Generally, the performance of ion osmotic energy storage devices is significantly influenced by environmental factors like temperature and humidity due to their impact on the physicochemical properties of the materials involved [45–47]. Temperature variations can alter the ionic conductivity and mobility within the electrolyte, affecting the rate and efficiency of ion transport. Humidity's impact on ion osmotic energy storage devices arises from its effect on the hydration layers around the ions in the electrolyte and electrode, which can change the diffusion dynamics of ions. The ion solvation in IOPS is provided by ionic liquids and is not disturbed by water. Figure S18a presents result that demonstrates IOPS's resistance to environmental humidity, confirming its electrical performance remains unaffected by moisture. Previously, we established the temperature dependence of the ionogel, noting that increases in ambient temperature significantly enhance the ion diffusion rate within IOPS. Notably, the device operates effectively across a wide temperature range, from − 10 to 80 °C, with increased temperatures significantly boosting the peak *J*_SC_ without substantially affecting the voltage (Fig. S18b). The design strategy of IOPS is versatile, making it suitable for fabricating devices with varying working areas. As shown in Fig. S18c, the peak *J*_SC_ increases with larger working areas.

### Working Mechanism of the IOPS

**Fig. 3:**
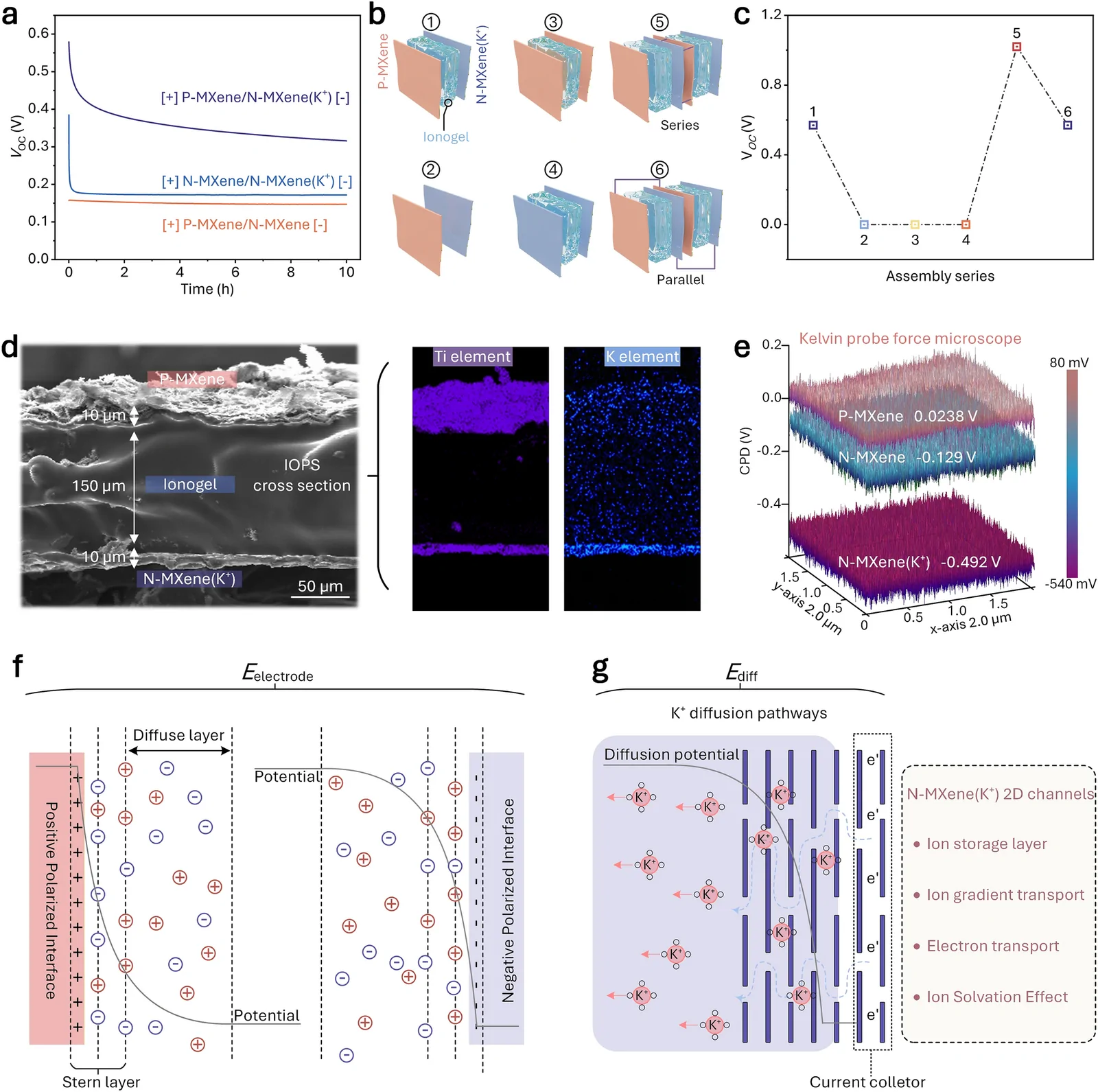
Working mechanism of the IOPS. **a** Voltage output of IOPS using different electrode combinations of P-MXene/N-MXene(K^+^) (purple), N-MXene/N-MXene(K^+^) (blue), and P-MXene/N-MXene (orange). **b, c** Initial voltage generated by the combination of various components of IOPS. **d** SEM cross-sectional image of the IOPS device and EDS spectra of important elements (Ti, K). **e** Relative surface potentials of P-MXene, N-MXene, and N-MXene(K^+^) measured by KPFM. **f** Schematic diagram of the principle of *E*_electrode_. **g** Schematic diagram of the principle of *E*_diff_. K^+^ diffuses through the solvated structure

While generally *V*_OC_ is the projection of the Fermi level difference in the electrolyte environment, its practical manifestation in the IOPS system emerges from the interplay between interfacial electrode potentials (*E*_electrode_) and ionic diffusion potentials (*E*_diff_). To decouple these synergistic contributions, we systematically investigated three archetypal electrode configurations through comparative *V*_OC_ measurements under controlled ion transport conditions. As shown in Fig. 3a, when the positive electrode of the original device [ +]P-MXene/N-MXene(K^+^)[ −] (purple line) was replaced with N-MXene, the initial *V*_OC_ showed 0.395 V and dropped rapidly within half an hour (blue line). We analyzed that N-MXene and N-MXene(K^+^) have similar functional group compositions. The K^+^ stored in N-MXene(K^+^) produces entropy-driven ion diffusion behavior under the influence of a resting-potential-mimetic mechanism, so an obvious potential (*E*_diff_) downward trend is generated in the first half an hour. The *V*_OC_ tends to stabilize after half an hour. This stable potential arises from the distinct Fermi energy levels (or work functions) of N-MXene and N-MXene(K^+^). Although both possess similar electronegative terminals, the pre-intercalation of K^+^ induces an electronic n-doping effect, shifting the Fermi level of N-MXene(K^+^) upwards relative to pristine N-MXene (a difference in surface chemical environment verified by XPS and zeta potential characterization). To further elucidate the devices' dynamic characteristics, AC electrochemical impedance spectroscopy (EIS) was conducted across a frequency range of 0.01 Hz to 0.1 MHz, evaluating heterogeneous charge transfer parameters. Nyquist plots in Fig. S19a − c show no clear semicircles in the high-frequency regions of the [ +]N-MXene/N-MXene(K^+^)[ −] and [ +]P-MXene/N-MXene(K^+^)[ −] devices. This may result from the lower R_s_ value leads to relatively fast ion transport at the negative electrode interface. Concurrently, diffusion control dominates the system and the charge transfer resistance (R_ct_) process is not significant. Especially in the low-frequency region, the semicircle is replaced by the oblique line of Warburg impedance. This feature also reflects the fast diffusion kinetics of K^+^. In addition, the Bode plot (Fig. S19d-f) shows that the minimum phase angle for [ +]P-MXene/N-MXene(K^+^)[ −] is − 54°, decreasing rapidly as frequency increases. The frequency at which the imaginary part is equal to the real part (phase angle of − 45°) is considered to be the cutoff frequency. Electrodes with higher cutoff frequencies exhibit faster charging and discharging capabilities. Figure S19f reveals that the electrode's cutoff frequency in this study is 17 Hz. The corresponding relaxation time constant of approximately 9.4 millis indicates the electrode's rapid charge transfer and enhanced power transfer capabilities.

In order to more intuitively observe the source of IOPS voltage output, Fig. 3b, c displays *V*_OC_ measurements for devices with various configurations, with Device 1 maintaining the original setup. Devices 2, 3, and 4 showed no electrical signals. These results further indicate that unreasonable structural distribution cannot produce any electrical output: Device 2 shows that both P-MXene and N-MXene(K^+^) have excellent electronic conductivity, and direct contact will lead to a short circuit; Device 3 shows that the lack of heterogeneous charge structure also cannot generate any electrical signal; Device 4 shows that the mutually offsetting ion diffusion effect will result in no electrical signal being generated. Therefore, only a properly structured distribution, such as in Device 1, can facilitate efficient ion-electron coupling, which is the primary reason for electricity generation. Devices 5 and 6 demonstrate the series and parallel *V*_OC_ configurations of IOPS, highlighting excellent device scalability.

To further explore the working mechanism of this novel type of IOPS power device, we used a variety of characterization techniques to analyze the ion evolution during the discharge process. In the previous section, we have verified through electrical testing that ion diffusion and the Fermi level difference of electrodes are the main driving forces for IOPS energy harvesting. EDS element mapping in Fig. 3d illustrates the distribution of titanium elements in the polarized MXene-based electrodes. After the device was short-circuited for 10 min to dissipate energy, it was clearly observed that the K element diffused from N-MXene (K^+^) to the ionogel, which verified the formation mechanism of the K^+^ diffusion current. Furthermore, ICP-OES provided direct evidence of ion transport. The K^+^ concentration in the ionogel after discharge of five independent devices was measured, increasing from 0 to an average of 7873 ppm (Fig. S20). In order to generate power output, gradient diffusion and directional transport of ions need to be converted into electron transport at the electrode surface. In traditional RED systems, this conversion is typically achieved through the redox reactions at Ag/AgCl electrodes [9, 21, 22, 48]. In hydrovoltaic devices, although the mechanism of current generation has not yet been fully explained, it is preliminarily believed to be achieved through mirror-electrons or image-electrons [43, 49–51]. In our IOPS system, since both N-MXene(K^+^) and P-MXene have excellent metallic conductivity, they can directly release and accept electrons to complete this process. Work function is an important term in solid physics and surface science, which represents the minimum energy required to remove an electron from the surface of a material. Specifically, the work function is the energy required to raise an electron from the Fermi level of a material to the vacuum level. When two different electrodes are assembled into a generator, the electrons of the electrode with a lower work function will spontaneously flow to the electrode with a higher work function. This is because electrons tend to occupy states with lower energy. This electron transfer generates a potential difference between the two electrodes until a state of equilibrium is reached. Kelvin probe force microscopy (KPFM) is a high-precision surface measurement technique that can measure local work function changes on the surface of a sample [52, 53]. It can indirectly calculate the work function of the sample surface by measuring the contact potential difference (CPD) between the sample surface and the conductive probe. Figure 3e shows the CPD landscape images of P-MXene, N-MXene, and N-MXene(K^+^) films within a 2.0 μm × 2.0 μm area, and presents area scan measurements of surface potential changes in the three MXene-based electrodes. The average measured CPDs are 0.0238, − 0.129, and − 0.492 V, respectively. Figure S21 provides the surface morphology images of the corresponding films. In addition, under ideal conditions, the CPD difference is highly positively correlated with the potential difference between the electrodes. This offers a direct method for estimating the *V*_OC_ of IOPS devices. By calculating the CPD differences between P-MXene, N-MXene, and N-MXene(K^+^), a positive correlation consistent with the initial *V*_OC_ of three types of IOPS devices is observed (minor discrepancies may arise from electrochemical effects, interfacial phenomena, and other corrective factors). This confirms the rational design of the IOPS. Furthermore, using a gold standard sample, we determined the KPFM probe's work function to be approximately 5.0095 eV (Fig. S22). Consequently, the work function of three MXene-based electrodes were calculated to be 5.033 eV for P-MXene, 4.881 eV for N-MXene, and 4.518 eV for N-MXene(K^+^) (Note S1.7). The APTES-derived silane terminals form O-Si bonds with hydroxylated MXene surfaces and introduce amine-containing terminal groups, which can alter the surface electrostatic potential and interfacial dipole/charge distribution; this change is consistent with the KPFM-observed increase in work function and the corresponding lowering of the Fermi level for P-MXene. Therefore, it is easy to discern their propensity to serve as either the positive or negative electrode in IOPS. P-MXene has a relatively high work function and is suitable as a positive electrode, which helps prevent excessive escape of electrons. N-MXene(K^+^) is very suitable as a negative electrode, which is conducive to releasing ions and providing sufficient electrons.

Based on the above observations, we propose the following mechanism for voltage generation driven by thermodynamic synergy. Upon device assembly, the ionogel forms intimate solid-state contact with the two polarized MXene electrodes. The significant difference in work function between P-MXene and N-MXene(K^+^) induces a potential bias at the device interfaces (Fig. 3f). Concurrently, the pre-loaded K^+^ within the N-MXene(K^+^) tends to dissociate from the interlayer reservoirs and migrate into the ionogel in a solvated state (Fig. 3g). Therefore, the initial open-circuit voltage (*V*_initial_) of the IOPS should be understood as the combined outcome of the electrode energy asymmetry and the diffusion of K^+^ driven by the activity gradient, rather than arising from a singular process. As the device progressively approaches equilibrium, the diffusion-related contribution diminishes, and the residual voltage is predominantly sustained by the inherent energy level difference between the two polarized MXene electrodes (for further discussion, please refer to Note S1.8).

### Multi-Scale Simulation

**Fig. 4:**
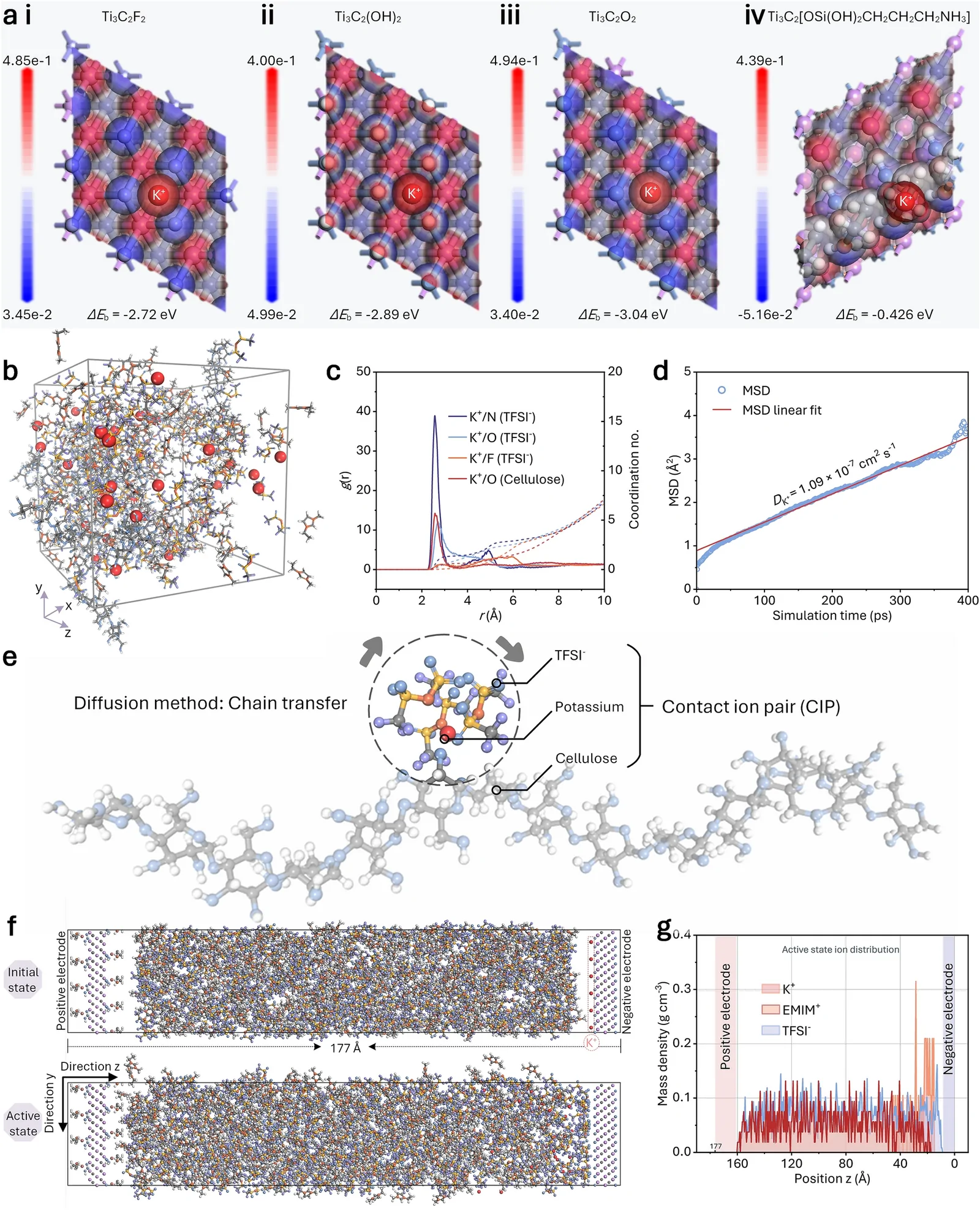
Working mechanism under theoretical simulation. **a (**i-iii) The ability of the molecular structures of three different terminal groups of N-MXene (Ti_3_C_2_F_2_, Ti_3_C_2_(OH)_2_ and Ti_3_C_2_O_2_) to interact with K^+^, and the molecular electrostatic potential energy surface. iv, The ability of the representative monodentate configuration of P-MXene (Ti_3_C_2_[OSi(OH)_2_CH_2_CH_2_CH_2_NH_3_) to interact with K^+^, and the molecular electrostatic potential energy surface. The scale unit is Hartree/e. **b** Snapshots obtained from MD simulations of ionogel (for specific parameters see Note S1.6).** c, d** Radial distribution function of K^+^, and K^+^ diffusion coefficient derived from MSD results. **e** The most likely solvation structure and transport mode of K^+^ in ionogel extracted from MD simulation.** f** Initial state model and discharge activation state (2 ps) model of IOPS simulated by MD. **g** Mass density distribution probabilities of K^+^, EMIM^+^, and TFSI.^−^ along the IOPS normal (z-axis)

To further elucidate the experimentally observed dual-contribution mechanism involving electrode potential and ion diffusion, we performed multi-scale simulations combining DFT and MD. First, we elucidated the microscopic origin of the Fermi level difference (*E*_electrode_) by analyzing the electronic interactions at the electrode terminals. MXene is characterized by a hexagonal unit cell structure with a well-defined number of layers. Figure S23 describes the construction of unit cell and supercell models without installed end-group configuration to avoid spurious interactions. For the ion terminal occupied sites in the MXene lattice, the optimized model of face-centered cubic (*fcc*) minimum energy configuration was used for calculation [54]. The fcc site is located at the hollow top composed of Ti elements, and no elements exist in the next layer. The MXene molecular structure used in this study can be simplified into four derivative forms. DFT calculations were employed to model the electrostatic potential (ESP) surfaces of N-MXene (with–F,– OH,–O terminals) and P-MXene (with–NH_2_-like monodentate terminals) (Fig. 4a). The ESP mapping reveals a stark contrast: N-MXene terminals exhibit deep blue/low-potential regions due to the high electronegativity of oxygen and fluorine atoms, confirming their "Negative Polarized" nature. In contrast, P-MXene displays red/high-potential regions, confirming its "Positive Polarized" nature. This intrinsic polarity difference is the root cause of the work function mismatch (*ΔΦ*) identified in the previous section. Binding energy (*ΔE*_b_) calculations further explain the EDL formation mechanism. As shown in Figs. 4a and S24, N-MXene exhibits a strong affinity for cations (e.g., strong K^+^–O interaction, *ΔE*_b_ =  − 3.04 eV), energetically favoring the adsorption of EMIM^+^ and K^+^ to form the negative-side EDL. Conversely, P-MXene shows weaker interaction with cations but is electrostatically prone to attracting anions (TFSI^−^). This selective adsorption tendency, dictated by the surface energetic landscape, spontaneously establishes the built-in electric field at the interface upon assembly, thereby validating the *E*_electrode_ contribution to the open-circuit voltage.

Simultaneously, the K^+^ concentration gradient establishes the thermodynamic foundation for the diffusion potential (*E*_diff_). Even in the static state, K^+^ ions exhibit a strong entropy-driven tendency to diffuse from the reservoir into the ionogel. To understand this process in an anhydrous environment, we analyzed the solvation dynamics. Binding energy calculations (Fig. S25) reveal a much stronger affinity of K^+^ for TFSI^−^ anions (− 470 kJ mol^−1^) compared to EMIM^+^ cations (− 104 kJ mol^−1^), governing the solvation structure. Unlike aqueous systems, radial distribution function (RDF) analysis (Fig. 4b, c) shows that K^+^ forms a unique Contact Ion Pair (CIP) structure [55]. The K^+^ solvation shell mainly comprises TFSI^−^ molecules, with average coordination number of 1.37 TFSI^−^ nitrogen per K^+^. In addition, the K^+^ solvation shell also includes 2.35 tightly double-layer coordinated TFSI^−^ oxygen and 0.65 Cellulose oxygen. This implies that K^+^ does not migrate as a naked ion but moves as a solvated cluster [K(TFSI)_x_]^(1−x)^. The mean square displacement (MSD) yields a diffusion coefficient of 1.09 × 10^−7^ cm^2^ s^−1^ (Fig. 4d). Notably, consistent with Manning's counterion condensation theory, the cellulose chains facilitate this transport by forming a charge layer that assists the ion dissociation [39, 56]. The cellulose network contributes to the ionogel beyond serving as a passive scaffold. During ionogel formation, [EMIM][TFSI] partially reconstructs the hydrogen-bond network of cellulose, converting the porous membrane into a continuous solid electrolyte while preserving its macroscopic shape. This reconstructed network provides mechanical integrity, adhesion, and resistance to interfacial delamination during deformation. At the same time, the retained ionic liquid supplies the ion-conducting phase, and the cellulose oxygen atoms participate in the local K^+^ solvation environment according to the RDF analysis. Therefore, the cellulose network helps couple mechanical stability with ionic conductivity by maintaining a continuous ionogel framework and assisting the solvation/transport environment for K^+^-containing clusters.

To elucidate the dynamic transport mechanism that converts this potential into current, we mapped the microscopic pathway and macroscopic flow. At the atomic level, the diffusion kinetics at the N-MXene/[EMIM][TFSI] interface follow a "hopping" mechanism (H_1_ → H_2_ → H_3_), as visualized by time-lapse trajectories (Fig. S26a, d). Initially, K^+^ is mainly coordinated with the anchor anion site at a distance of 2.4 Å from MXene-O (state H_1_). Subsequently, K^+^ undergoes an intermediate state H_2_, in which K^+^ dissociates from the nitrogen center part of the anion and jumps to the vicinity of the adjacent EMIM molecule. In the third step, K^+^ migrates along the molecular structure, re-forming K–O coordination at a distance of 2.2 Å (state H_3_). Figure S26b clearly shows the diffusion trajectory of K^+^ in three-dimensional space, and its migration barrier is about 0.985 eV (Fig. S26c). On a macroscopic scale, MD simulations of the discharge process (Fig. 4f, g and Movie S1, Note S1.9) visualize the collective carrier flow: driven by the electrochemical potential difference, K^+^ and EMIM^+^ migrate towards the positive electrode, while TFSI^−^ anions counter-migrate towards the negative electrode. This bidirectional transport confirms that the IOPS current is sustained by the synergistic release of K^+^ from the reservoir and the global re-balancing of electrolyte ions.

### Extensible Integration of IOPS

**Fig. 5:**
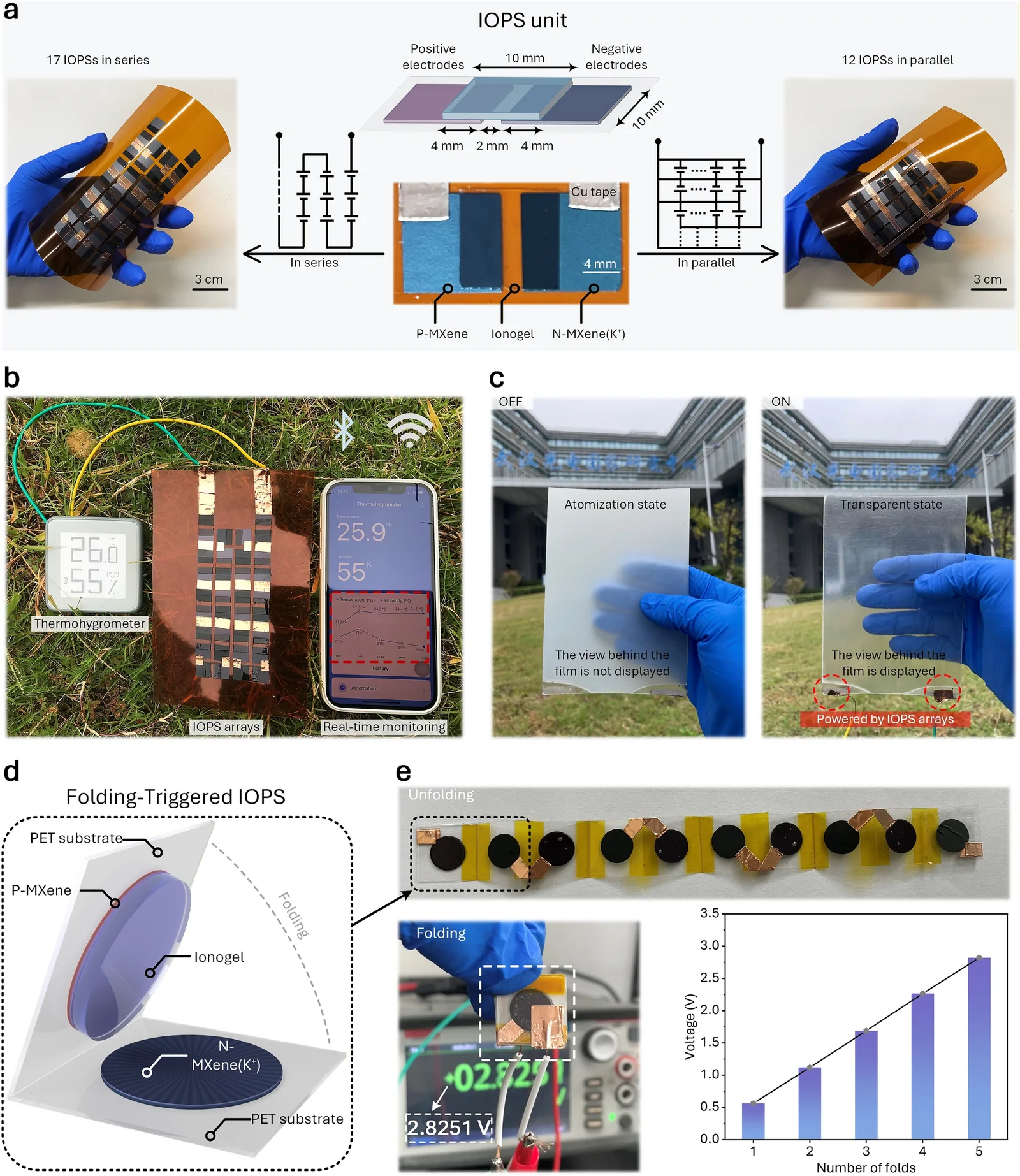
Extensible integration of IOPS. **a** Assembly and optical images of an IOPS unit, and schematic diagram of scalable integration of serial and parallel IOPS arrays. **b** IOPS array powers the thermohygrometer to monitor the ambient temperature and humidity in real time. **c** IOPS arrays powers the ECD to transform it from an atomization state to a transparent state. **d** Design concept diagram of the folding-trigger IOPS.** e** Optical schematic diagram of the unfolding and folding of 5 folding trigger IOPS arrays. The *V*_OC_ of the integrated IOPS units achieves a nearly linear output

Our comprehensive study of the IOPS mechanism has enabled the large-scale design and integration of IOPS units, achieving high-performance power output. Using appropriate series and parallel connections allows us to flexibly control the output voltage and current of the IOPS arrays. Figure 5a illustrates the modular design concept of the IOPS arrays. Here, we propose a new design scheme for IOPS units. Unlike the sandwich structure initially demonstrated, this design secures P-MXene, N-MXene(K^+^), and ionogel to the substrate, enabling more diverse circuit combinations. Figure 5a (middle) shows the dimensions of a single IOPS, with electrode spacings of 2 mm and a contact area of 4 mm × 10 mm between the ionogel and each electrode. Figure 5a (left and right) shows the series connection of 17 IOPS units and the parallel connection of 12 IOPS units assembled on the PI substrate. This configuration generates a *V*_OC_ of approximately 8.6 V and a maximum *I*_SC_ of 3.1 mA (Movie S2). This confirms the excellent scalability of the IOPS arrays. Additionally, the energy stored by the IOPS arrays can sufficiently power various commercial electronic products, including an ink screen thermohygrometer. Owing to its excellent environmental stability, the IOPS can power the thermohygrometer outdoors, as depicted in Fig. 5b. Real-time collection of ambient temperature and humidity is achieved. The IOPS arrays were further utilized to power an electrochromic device (ECD), as shown in Fig. 5c. When disconnected from the IOPS arrays, the ECD remains in an atomized state, making the scenery behind it almost invisible. When voltage is applied via the IOPS, the ECD becomes transparent. In addition, since entropy increase-driven ion diffusion occurs immediately after the IOPS device is assembled, it causes the ion diffusion energy in the device to be consumed in advance. To address this issue, we also designed a folding-triggered IOPS that does not dissipate energy when separated, as illustrated in Fig. 5d. In the unfolded state, the ionogel contacts only P-MXene, which does not store ions. Upon folding, it immediately generates power output. Figure 5e shows a folding-triggered IOPS with five units in series, which produces a *V*_OC_ of approximately 2.83 V when folded. Furthermore, the *V*_OC_ from the integrated IOPS unit exhibits a nearly linear output. As a proof of concept, we completed the power supply of the IOPS arrays for various application scenarios, underscoring the robust design and broad applicability of IOPS.

## Conclusions

In summary, the natural mechanisms of resting potential in biological systems have greatly inspired our comprehensive design strategy. In this study, by leveraging the unique interfacial energetic alignment properties of polarized MXene, we successfully emulated transmembrane ion transport at resting potential, enabling the development of an all-MXene solid-state iontronic osmotic power source. Through innovative working mechanisms and device architecture, the IOPS achieves a peak power density of 17.5 μW cm^−2^ and a volumetric power density of 1030 μW cm^−3^, with stable performance over 50 charge–discharge cycles. This performance far exceeds that of existing ion osmotic power source. More specifically, the key groundbreaking features of our study are as follows. (1) Working Mechanism. Unlike osmotic power sources that rely solely on ion gradient transport within stacked cells to generate electricity, the working mechanism of IOPS is the thermodynamic synergy of two simultaneous processes: the substantial energetic asymmetry between the two polarized MXene electrodes is consistent with asymmetric interfacial charge redistribution and EDL-like polarization at the electrode–ionogel interfaces, which contributes to the built-in interfacial potential; meanwhile, the solvated cations dissociate from the negative electrode reservoir and undergo diffusion dynamics under concentration gradient. (2) Device structure. By fully utilizing the ion–electron coupling properties of MXene, the functions of electrodes, current collectors, ion-selective layers, and ion reservoirs are highly integrated into MXene. This novel design enables the IOPS device to be assembled into a compact osmotic power supply using only three building blocks. Compared with similar studies (which typically require 4–7 building blocks), the assembly complexity and number of parts are significantly reduced. (3) Application paradigm. Thanks to the novel architecture and working mechanism, IOPS can easily achieve customized and scalable integration. IOPS arrays can efficiently power commercial electronic devices and show great potential in portable fast-response power systems.

## Supplementary Information

Below is the link to the electronic supplementary material.

Supplementary file1 (DOCX 3701 KB)

Supplementary file2 (MP4 10350 KB)

Supplementary file3 (MP4 12211 KB)
