# Supplementary material for: Resting-Potential-Inspired Solid-State Iontronic Osmotic Power Source Enabled by Polarized MXene
Source: Nanomicro Lett. 2026 Jul 22;18:449. doi: 10.1007/s40820-026-02310-9 (PMC13391481; doi:10.1007/s40820-026-02310-9)
Supplement: Supplementary file 1 — Supplementary file1 (DOCX 3701 KB) [file 40820_2026_2310_MOESM1_ESM.docx]

Supporting Information for

**Resting-Potential-Inspired Solid-State Iontronic Osmotic Power Source Enabled by Polarized MXene**

Ziqi Ren^1,2,3^, Long Zhang^1^, Qixiang Zhang^1^, Jianyu Yin^1^, Mingfang Deng^1^, Xubin Zhou^1^, Qianqian Yao^1^, Songzhan Li^4^, Yihua Gao^1^, Zhen Zhang^2,3,^* and Nishuang Liu^1,^ *

^1^ School of Physics & Wuhan National Laboratory for Optoelectronics (WNLO), Huazhong University of Science and Technology (HUST); Wuhan 430074, P. R. China

^2^ Department of Applied Chemistry, School of Chemistry and Materials Science, University of Science and Technology of China (USTC); Hefei 230026, P. R. China

^3^ State Key Laboratory of Bioinspired Interfacial Materials Science, Suzhou Institute for Advanced Research, University of Science and Technology of China (USTC); Suzhou 215123, P. R. China

^4^ School of Electronic and Electrical Engineering, Hubei Collaborative Innovation Center of Textile Industrial Chain Generic Technology, Wuhan Textile University (WTU), Hubei, 430200, P. R. China

*Corresponding authors. E-mail: zhenzhang@ustc.edu.cn (Zhen Zhang); nishuang_liu@foxmail.com (Nishuang Liu)

**S1 Supplementary Text**

***S1.1 Finite element simulation***

Multiphysics simulations of ionic currents in IOPS under dynamic conditions were performed based on Poisson and Nernst-Planck models. The coupling of the controlling Poisson and Nernst-Planck equations, with appropriate boundary requirements, is shown below:

$$\begin{aligned} \nabla^{2}\varphi= -\frac{F}{\varepsilon}\sum z_{i}c_{i}\#\left( S1 \right) \end{aligned}$$

$$\begin{aligned} j_{i}= D_{i}\left( \nabla c_{i}+ \frac{z_{i}Fc_{i}}{RT}\nabla\varphi\right)\#\left( S2 \right) \end{aligned}$$

$$\begin{aligned} \nabla j_{i}=0\#\left( S3 \right) \end{aligned}$$

Here, the physical quantities *ϕ*, *F*, *ε*, *z*, *c*, *D*, *j*, *R*, and *T* represent the electric potential, Faraday constant, dielectric constant, valence of ionic species, ion concentration, diffusion coefficient, ion flux, universal gas constant, and absolute temperature, respectively. The dielectric constant and diffusion coefficient were estimated based on previous reports. The dielectric constant was estimated to be 15. The diffusion coefficient of the ionic liquid was set to 0.076 × 10^−9^ m^2^ s^-1^ [S1, S2]. The IOPS simplified base model was set according to the thickness of the SEM cross-sectional image, with a model width of 100 μm, of which the MXene height was 10 μm and the ionogel height was 150 μm. Other detailed setting parameters are shown in Table S2. The model was run under dynamic conditions for 4 ps with a time step of 10 fs. The boundary conditions of the IOPS potential are given by Gauss's law.

$$\begin{aligned} \boldsymbol{n}\cdot\nabla\varphi= -\frac{\tau}{\varepsilon}\boldsymbol{\#}\left( S4 \right) \end{aligned}$$

Where *τ* represents the surface charge density. The ions flux in steady state has the zero nomal components at boundaries:

$$\begin{aligned} \boldsymbol{n}\cdot j=0\boldsymbol{\#}\left( S5 \right) \end{aligned}$$

***S1.2 N-MXene(K^+^) and P-MXene preparation scheme notes***

**N-MXene(K^+^) note:** The N-MXene(K+) membrane was prepared by immersing a self-supporting N-MXene membrane in a high-concentration KOH solution and then sealing it. The reasons why KOH is used as the ion intercalation solution are: i) the alkalization process of KOH can introduce more oxygen-containing groups at the end of MXene, thereby improving the adsorption capacity of potassium ions, reducing the diffusion energy barrier, and accelerating ion diffusion; ii) the KOH alkalization process does not introduce other impurity anions; iii) the smaller hydration radius of K^+^ helps it to enter the interlayer space of 2D MXene more easily.

**P-MXene note:** Although reports have shown that MXene can be successfully modified to carry a positive charge, this electrostatic adsorption method introduces additional polymer chains [S3, S4]. Therefore, in our work, a method of directly positively electrifying the MXene terminals was used. APTES is a commonly used silane coupling agent. The silanol groups generated in water are highly reactive and easily form O−Si covalent bonds with hydroxyl groups on the surface of nanomaterials. Therefore, we used APTES to react with MXene nanosheets under acidic conditions and successfully prepared positively electrified P-MXene membranres by vacuum-assisted filtration.

***S1.3 Quantification of ion selectivity of P-MXene and N-MXene membranes***

P-MXene membranes and N-MXene membranes have 2D nanofluid channels with positive and negative charges, respectively, so that anions (cations) driven by the chemical potential gradient on both sides of the membrane spontaneously diffuse from the high concentration side to the low concentration side. KCl solution is usually used to calibrate the ion transfer number because K^+^ (1.96 × 10^−9^ m^2^ s^−1^) and Cl^-^ (2.03 × 10^−9^ m^2^ s^−1^) exhibit similar ion diffusion coefficients. This similarity results in symmetrical ion flow during osmotic, facilitating the creation of a simplified model for theoretical analysis. For a given concentration gradient, the ion transfer number (*t*) can be calculated as follows:

$$\begin{aligned} t= \frac{1}{2}\left( \frac{V_{\mathrm{diff}}}{\frac{RT}{zF}\ln\frac{\gamma_{c_{H}}c_{H}}{\gamma_{c_{L}}c_{L}}}+1 \right)\#\left( S6 \right) \end{aligned}$$

Among them, *V*_diff_, *R*, *T*, *F*, *z*, *γ*, and *c* represent osmotic potential, universal gas constant, absolute temperature, Faraday constant, charge number, ion activity coefficient, and ion concentration, respectively. According to the given concentration gradient, the anion transfer number *t*_Cl-_ of P-MXene is 0.750, and the cation transfer number *t*_K+_ of N-MXene is 0.972.

***S1.4 XRD pattern analysis*** *(****Fig. S9)***

The XRD pattern indicates that the (002) Bragg peak of P-MXene shifted from 6.1° in original N-MXene to 5.2°, reflecting changes in the space group. This shift suggests that the interlayer spacing expanded from 14.5 Å to 17.0 Å, primarily due to a pillaring effect from APTES-modified -[OSi(OH)_2_CH_2_CH_2_CH_2_NH_3_] functional groups causing 2D interlayer expansion. The shift of the peak of N-MXene(K^+^) to 6.7° indicates that its interlayer spacing is reduced to 13.0 Å, which is due to the electrostatic adsorption and close coordination of K-O bonds caused by the intercalation of potassium ions at the nanoscale, resulting in the collapse effect of the nanosheet layer.

***S1.5 XPS spectrum analysis*** *(****Fig. S10)***

Ti2p fine spectrum. By comparing the Ti2p spectra of N-MXene and N-MXene (K^+^), it is proved that alkalization-assisted K^+^ intercalation changes the interface oxidation state of the original MXene. The Ti 2p spectrum of N-MXene can be fitted into three groups of peaks at 455.2 and 461.2 eV, 456.3 and 462.4 eV, 459.3 and 465.3 eV, corresponding to Ti−C, Ti−F/Ti−O−F and titanium oxides, respectively. The titanium oxide peak of N-MXene (K^+^) after alkalization K^+^ intercalation is enhanced and moves to high energy, indicating that alkalization assists the formation of more oxidation states and potassium ion interactions. This increased oxidation state and interaction can cause the binding energy of Ti 2p_3/2_ and Ti 2p_1/2_ to shift to the high energy direction.

C 1s fine spectrum. By comparing the C1s spectra of N-MXene and N-MXene (K^+^), the strong interaction between K^+^ and the surface electrons of MXene can be explained. By observation, N-MXene(K^+^) has two more peaks at 292.7 eV and 295.4 eV than N-MXene. The peak at 295.4 eV indicates that potassium ions are embedded in the defect sites or interlayer structures of MXene materials, forming a state of strong charge transfer, which may be described as K^+^-C. The peak at 292.5 eV can be described as O–C–K^+^, indicating that potassium ions interact electrostatically with oxidized carbon groups.

O1s fine spectrum. By comparing the O1s spectra of N-MXene and P-MXene, the formation of silyl ether bond structure can be explained. The peak at 532.7 eV of P-MXene shifts to 531.5 eV in the high energy direction, indicating that the modification of APTES forms new silyl ether bonds on the surface of MXene. Moreover, the surface of the modified P-MXene undergoes charge rearrangement, and the additional electron donor provided by -NH_3_ may affect the electron density of the surface oxidation state, thereby causing the shift of the O 1s peak.

***S1.6* *FT-IR spectroscopy analysis*** *(****Fig. S11)***

The FT-IR spectrum of the original N-MXene shows characteristic peaks at 3440 cm^−1^, 1631 cm^−1^, 1017 cm^−1^ and 552 cm^−1^, which are analyzed one by one.

3440 cm^−1^: This peak is usually related to the O−H stretching vibration of adsorbed water molecules (H_2_O) or hydroxyl groups (−OH) on the MXene surface.

1631 cm^−1^: Generally related to the bending vibration of adsorbed water or the bending vibration of hydroxyl groups (−OH).

1017 cm^−1^: Corresponds to the stretching vibration of the C−F bond of the MXene molecule.

552 cm^−1^: This is a typical Ti−O vibration peak of the MXene molecule.

For N-MXene(K^+^), it is clearly found that the Ti−O vibration peak is red-shifted (moved to a lower wave number). This is because the intercalation of K^+^ causes a change in the electronic structure of the Ti−O bond. After the introduction of potassium ions, electrostatic interactions are formed with oxygen functional groups on the surface of MXene, and part of the electron density is transferred to the potassium ions, resulting in an increase in the polarity of the Ti−O bond. For P-MXene, new characteristic peaks appear, corresponding to Si−O stretching vibration at 872 cm^−1^ and N-H bending vibration at 1645 cm^−1^, respectively, which are signs of successful grafting of P-MXene.

***S1.7 Calculation of the probe work function by CPD***

The work function of the probe is calculated using the contact potential of the gold standard sample:

$$\begin{aligned} \Phi_{\mathrm{tip}}= \Phi_{\mathrm{Au}}-e \times\mathrm{CPD}\#\left( S7 \right) \end{aligned}$$

Where *Φ*_tip_ is the work function of the probe, *Φ*_Au_ is the work function of gold (5.1 eV), and e is the charge of the electron. The contact potential difference of the gold standard obtained by the test is 0.0905 V. Therefore, the work function of the probe is about 5.0095 eV.

The work functions of the three MXene-based electrode samples can be calculated using the gold standard sample:

$$\begin{aligned} \Phi_{\mathrm{sample}}= \Phi_{\mathrm{tip}}-e \times\mathrm{CPD}\#\left( S8 \right) \end{aligned}$$

"Sample" refers to the work function of the sample, "tip" to that of the probe, and "*e*" to the electron charge.

***S1.8 Voltage generation mechanism***

Unlike traditional aqueous reverse electrodialysis devices, the initial open-circuit voltage (*V*_initial_) in this anhydrous system originates from the inherent electrode potential and ion diffusion. Its approximate calculation formula can be written in the following form:

$$\begin{aligned} V_{\mathrm{initial}}\approx\frac{\Phi_{P-MXene}-\Phi_{N-MXene\left( K^{+} \right)}}{e}+{V_{diff}+V}_{\mathrm{corrective}}\#\left( S9 \right) \end{aligned}$$

Where *Φ*_P-MXene_ and *Φ*_N-MXene(K+)_ are the work functions of the positive (P-MXene) and negative (N-MXene(K^+^)) electrodes, respectively, and e is the elementary charge. Where *V*_diff_ represents the diffusion potential and *V*_corrective_ represents the correction factors arise from electrochemical effects, interfacial phenomena, and other factors.

***S1.9 Molecular dynamics simulation***

MD of the electrolyte structure: The simulation box contained 30 K^+^, 4 cellulose chains with a degree of polymerization of 15, and 112 [EMIM][TFSI] molecules, corresponding to the experimental value of the mass percentage of the ionogel (experimental methods in the main text). First, 100 Forcite dynamics were performed at 298 K to randomize the initial shape of the molecules. Then, an initial energy minimization was performed using the COMPASS Ⅲ force field at 298 K to obtain the ground state structure. After this, MD simulations were performed using the Nose-Hoover thermostat, and the system performed 500 ps of dynamics at 298 K in the NVT ensemble with a time step of 1 fs. The radial distribution function was obtained using the visualization method.

MD of the ionic distribution in the IOPS energy dissipation state (neglecting the influence of the cellulose chain): The simulation box contained 36 K^+^, 400 [EMIM][TFSI] molecules, and the 11 × 11 supercell structures of Ti_3_C_2_[OSi(OH)_2_CH_2_CH_2_CH_2_NH_3_] and Ti_3_C_2_O_2_ were used as the positive and negative electrodes, respectively. The force field parameters of MXene refer to some previous works [S5, S6]. In the simulation system, the MXene molecular structure is always kept fixed. 400 [EMIM][TFSI] have been pre-initialized to obtain the ground state structure. The MD system uses the NVT ensemble and runs at 298 K for 2 ps (time step is 1 fs). Use visualization methods to obtain the mass density distribution function.

COMPASS III was adopted here because it is an ab initio–derived condensed-phase force field with parameter coverage extended to ionic liquids, while the COMPASS family has also been shown to reasonably reproduce structural properties of cellulose systems; therefore, it is a practical choice for describing the relative ion-solvation and diffusion behavior in the present MXene/[EMIM][TFSI]/cellulose multiphase model [S7-S9].

**S2 Supporting Figures**


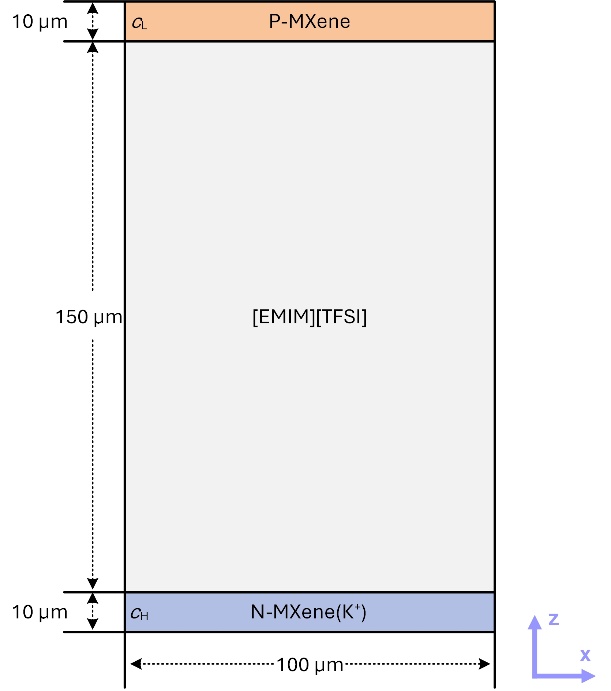


**Fig. S1** Finite element simulation model of the IOPS device (drawn to scale).

**
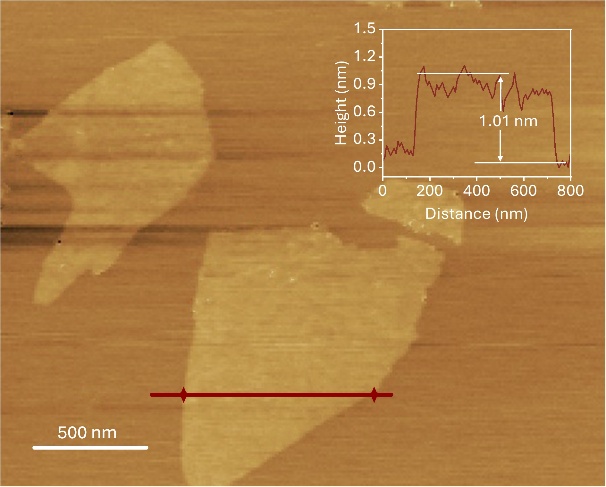
**

**Fig. S2 AFM characterization of N-MXene.** Inset shows the size information of N-MXene. Scale bar, 500 nm.


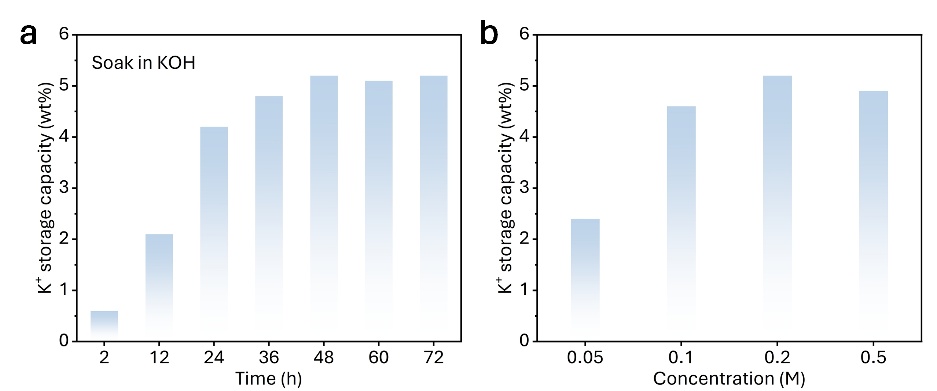


**Fig. S3 Comparative test of alkalization-assisted intercalation of N-MXene by KOH solution.** a, K^+^ intercalation mass percentage of N-MXene after soaking in 0.2 M KOH for different time (relative to the original mass of N-MXene). b, K^+^ intercalation mass percentage of N-MXene after soaking in KOH of different concentrations for 48 h (relative to the original mass of N-MXene).


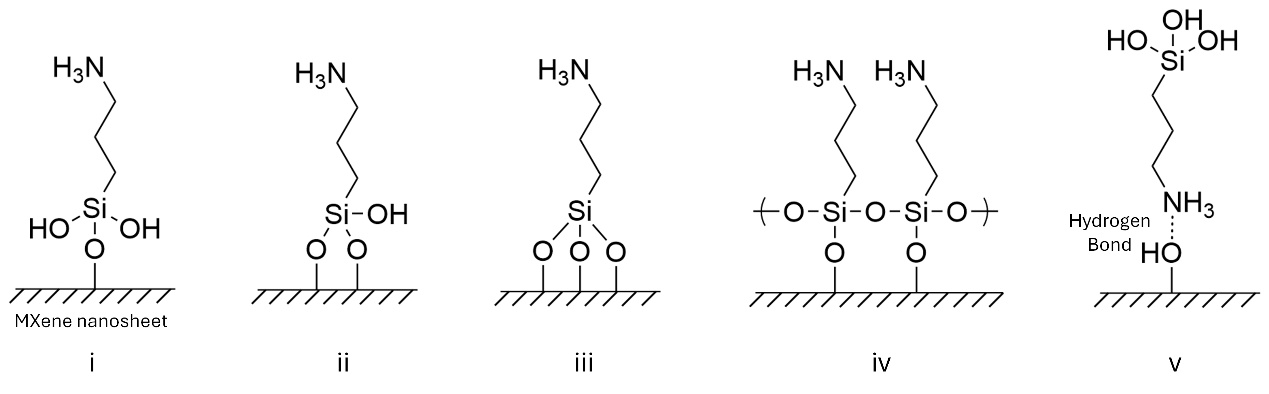


**Fig. S4 Schematic representation of the grafting modes of APTES on the surface of MXene nanosheets.** Figures i to iii show the grafting modes of P-MXene monodentate, bidentate and tridentate, respectively. Figures iv and v show lateral polymerization and hydrogen bonding of P-MXene terminal groups, respectively.


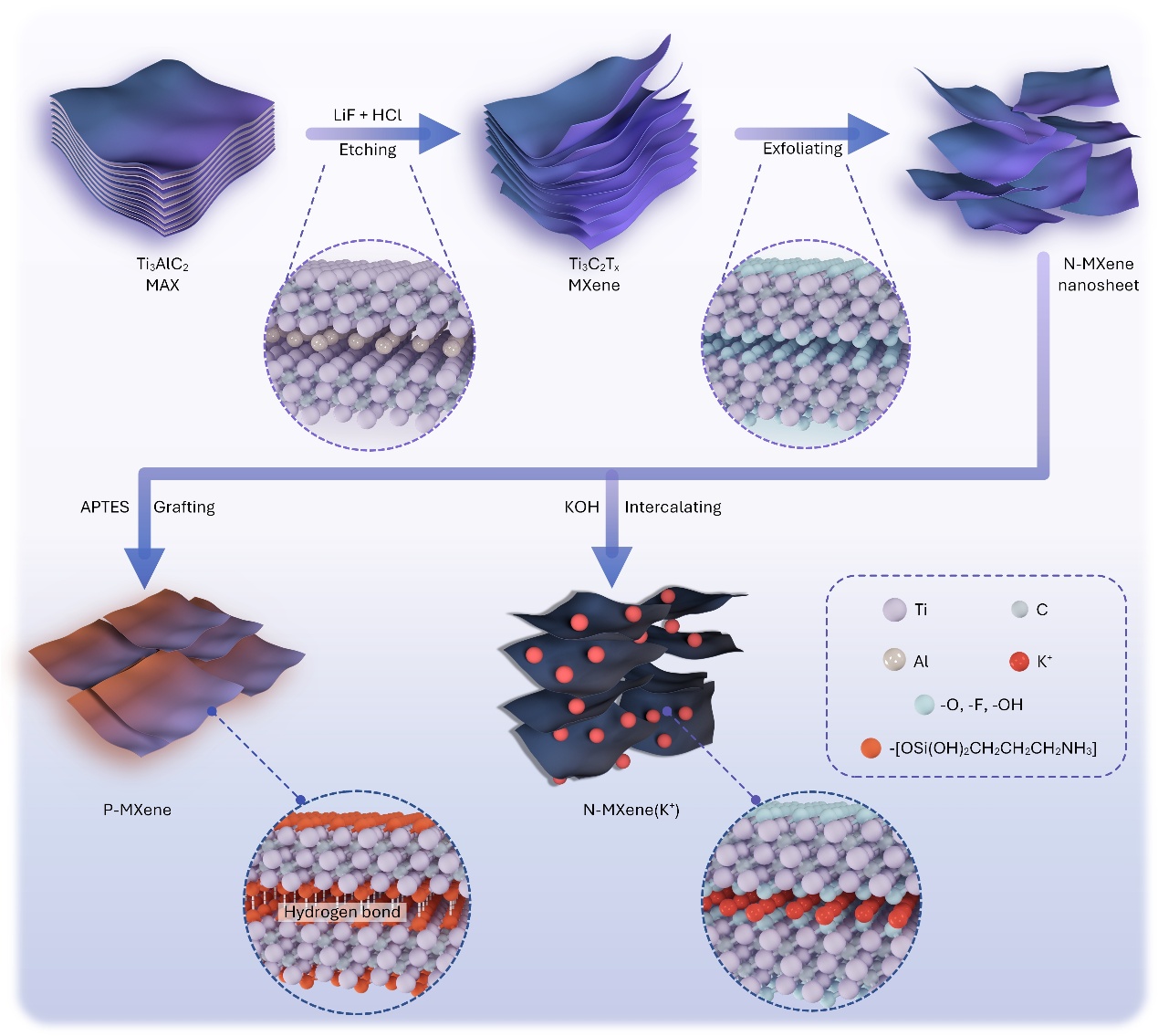


**Fig. S5 Schematic diagram of the preparation scheme of N-MXene, N-MXene(K^+^) and P-MXene.** The circular inset shows the detailed molecular structure information.


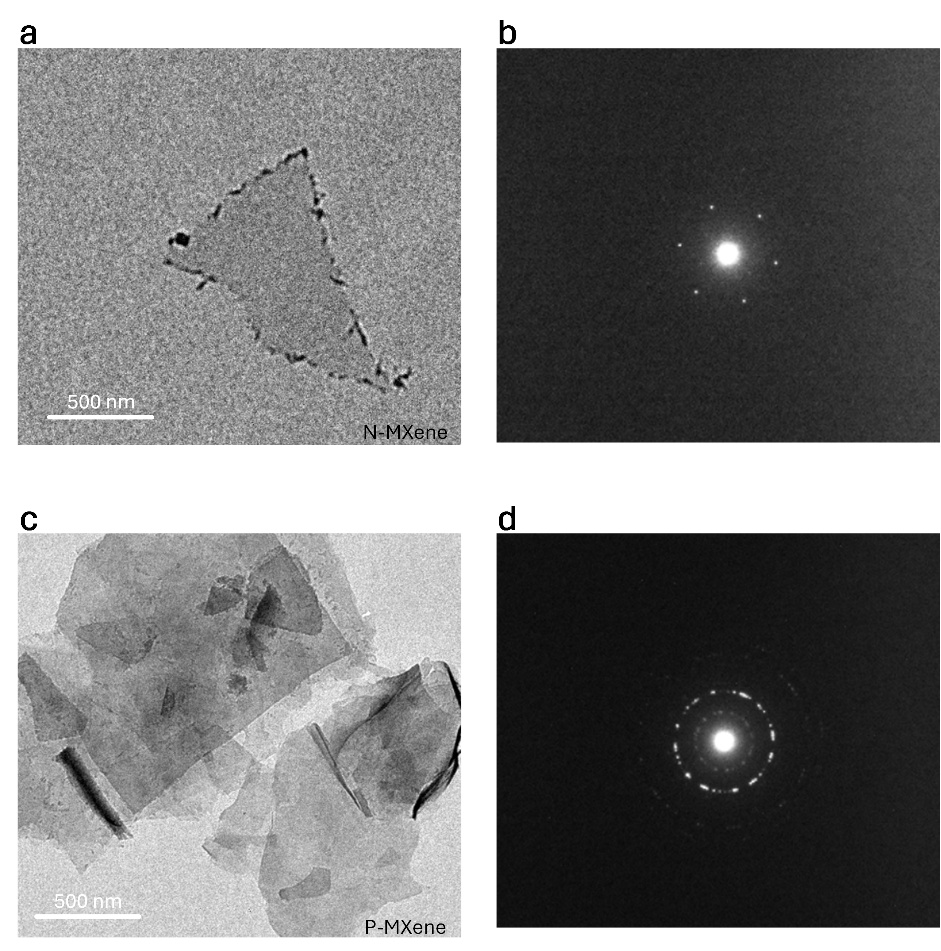


**Fig. S6 TEM characterization of N-MXene and P-MXene.** a and b, TEM image and SAED spectrum of N-MXene (single crystal). c and d, TEM image and SAED spectrum of P-MXene (polycrystalline). Scale bar, 500 nm.


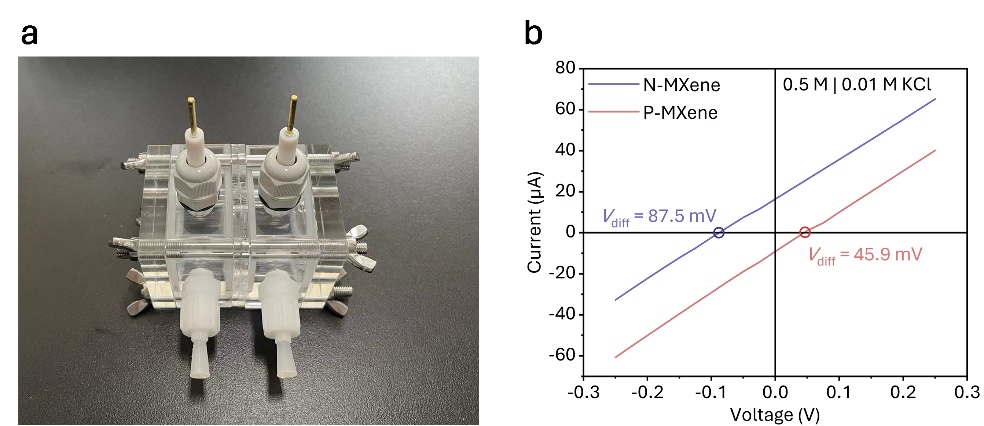


**Fig. S7** **Transmembrane ion transport properties.** a, Optical photograph of the transmembrane ion transport test device, with N-MXene or P-MXene membrane sandwiched in the middle of an H-type double-chamber electrochemical cell. b, *I* − *V* curve characteristics of N-MXene and P-MXene membranes recorded using Ag/AgCl electrodes containing salt bridges.


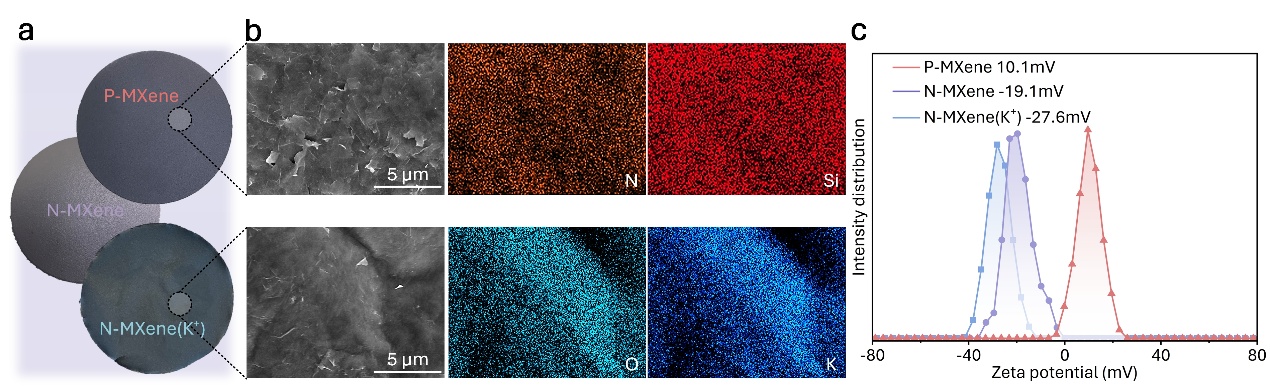


**Fig. S8 Characteristics of N-MXene, N-MXene (K^+^) and P-MXene membranes and the zeta potential of their solutions.** a, Optical photos of N-MXene, N-MXene(K^+^) and P-MXene membranes under natural light conditions. b, SEM surface morphology images of P-MXene membranes and N-MXene(K^+^) membranes and EDX mapping of typical elements (N, Si, O, K). c, Zeta potential of aqueous dispersions (0.1 mg mL^-1^) of N-MXene, N-MXene(K^+^) and P-MXene.


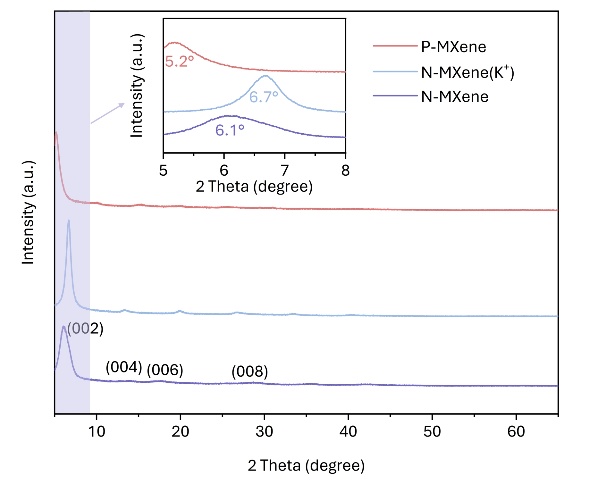


**Fig. S9 XRD characteristics of N-MXene, N-MXene(K^+^) and P-MXene membranes.** The inset shows the characteristic peaks of the (002) lattice planes of the three MXenes.


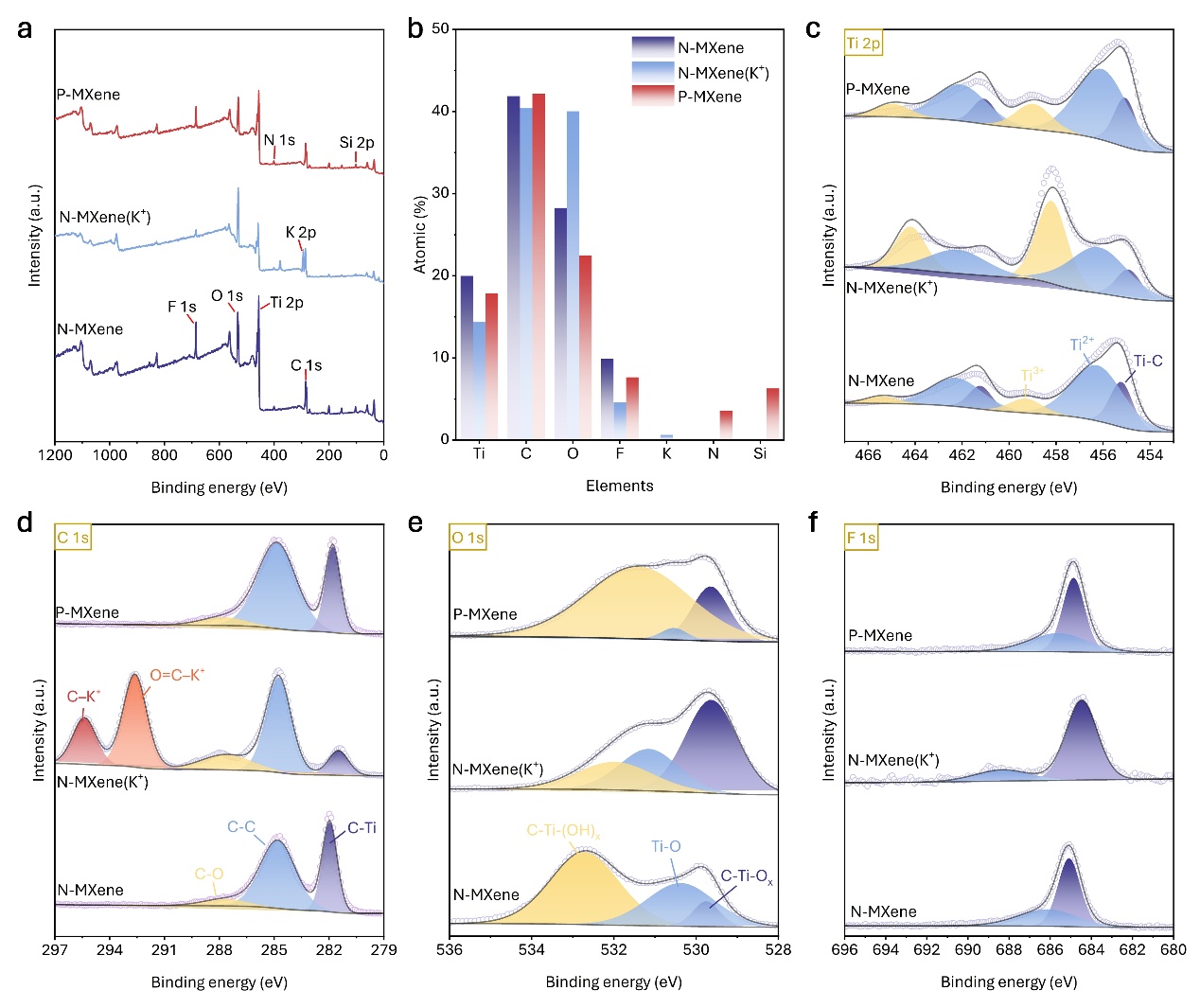


**Fig. S10** **XPS characteristics of N-MXene, N-MXene(K^+^) and P-MXene.** a, XPS scanning total spectrum of three MXenes. b, Characteristic element content of three MXenes. Ti 2p core level spectrum (c), C 1s core level Spectrum (d), O 1s core level spectrum (e) and F 1s core level spectrum (f).


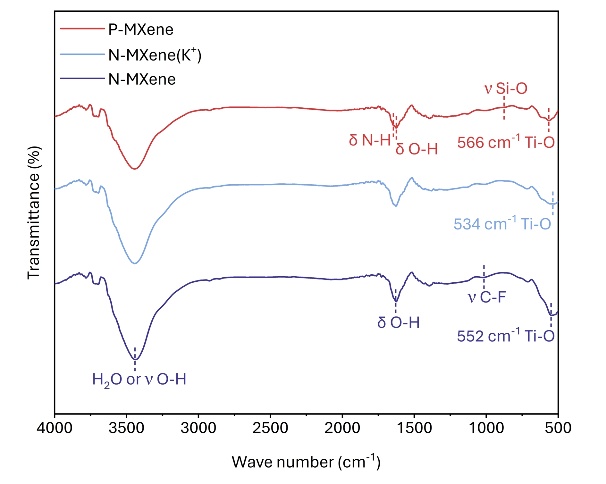


**Fig. S11 FT-IR characteristics of N-MXene, N-MXene(K^+^) and P-MXene.** The typical characteristic peaks of N-MXene, N-MXene(K^+^) and P-MXene are marked in the Figure. For example, Ti-O vibration peak, O-H bending vibration, O-H stretching vibration, C-F stretching vibration, etc.


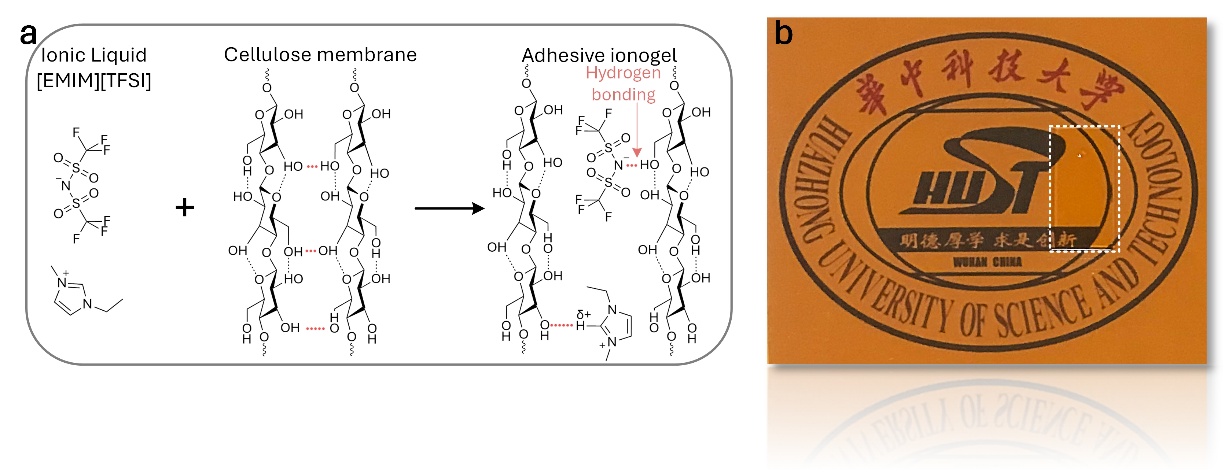


**Fig. S12 The formation mechanism of adhesive ionogel.** a, Ionic liquid [EMIM][TFSI] partially reconstructs the hydrogen bonds of cellulose membrane, causing it to directly form ionogel. b, Optical image of ion gel placed on PI substrate.


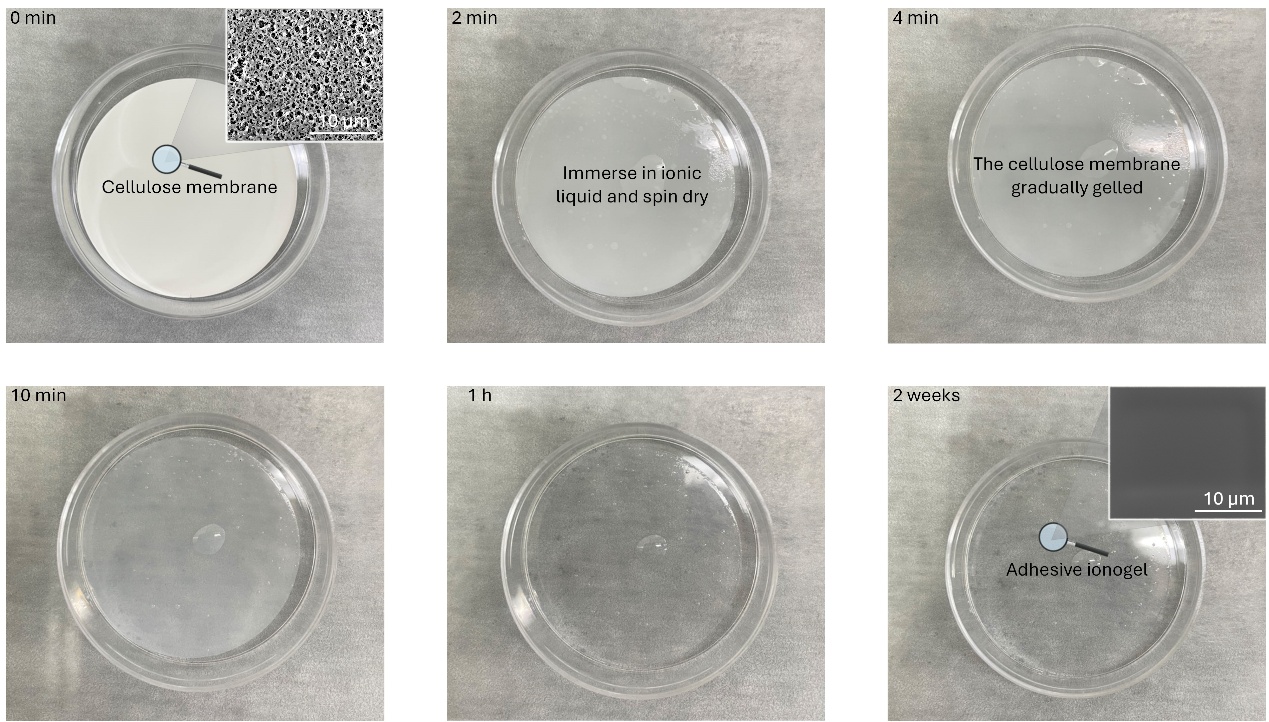


**Fig. S13 Morphological transformation of adhesive ionogel over time.** The figure shows that after immersion in ionic liquid, some hydrogen bonds of cellulose membrane are reconstructed, thus gradually transforming from a porous fiber structure to a uniform ionogel. The SEM surface morphology is shown in the upper right corner of the first and last images. Scale bar, 10 μm.


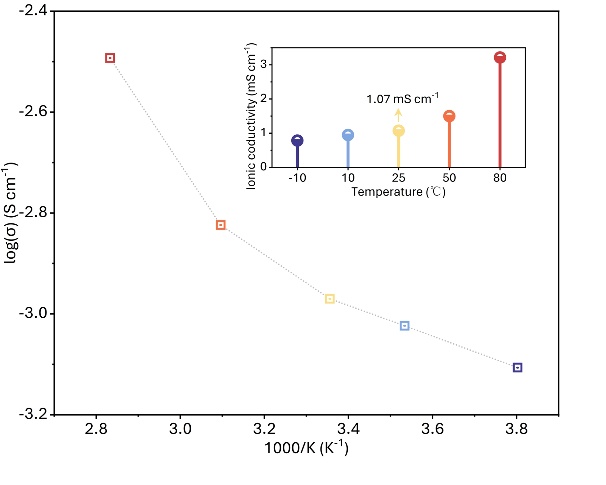


**Fig. S14 The conductivity-temperature dependence of ionogel.** As the temperature increases, the conductivity of ion gel further increases.


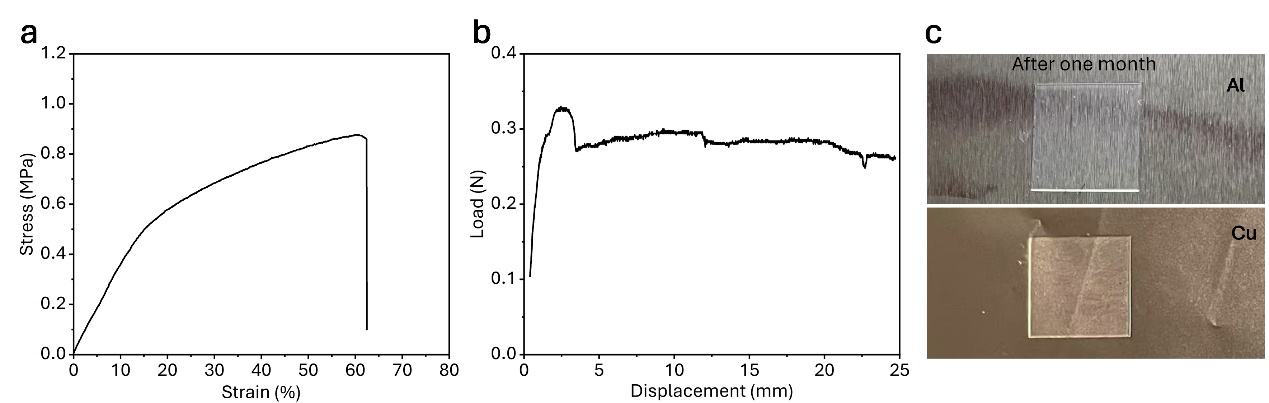


**Fig. S15 Mechanical properties and stability of ion gel.** a, Stress-strain curve of ionogel. b, Adhesion of ion gel (Since MXene-based membrane cannot withstand adhesion test, PI film is used as the test sample here). c, Optical photo of ion gel placed on aluminum foil and copper foil one month later.


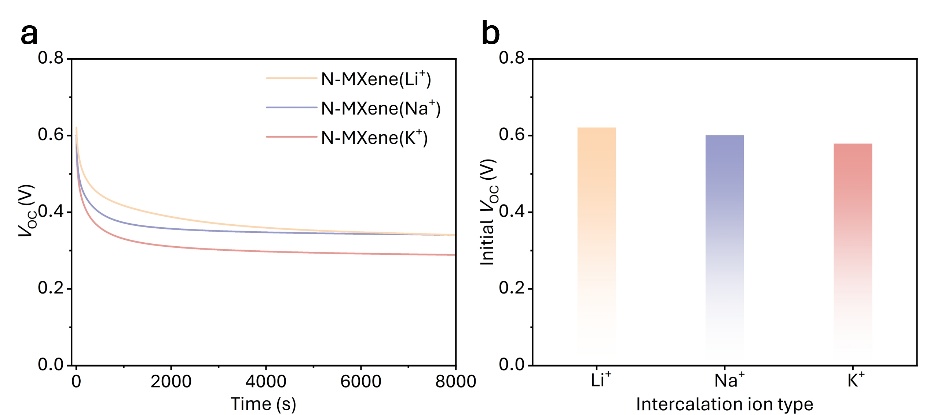


**Fig. S16 Electrical output performance of IOPS after different alkali metal ions (Li^+^, Na^+^, K^+^) are inserted into N-MXene.** a, Time-voltage curves of replacing the IOPS anode with N-MXene(Li^+^), N-MXene(Na^+^) and N-MXene(K^+^), respectively. b, Comparison of the initial voltages of the three IOPS devices.


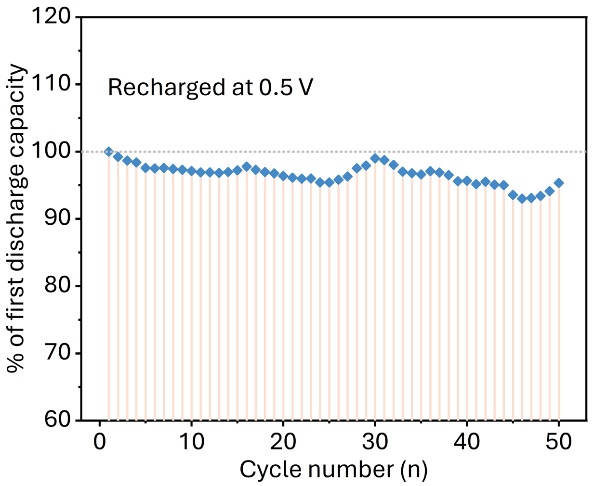


**Fig. S17 Lollipop plot of the normalized integral of the discharge curve.**


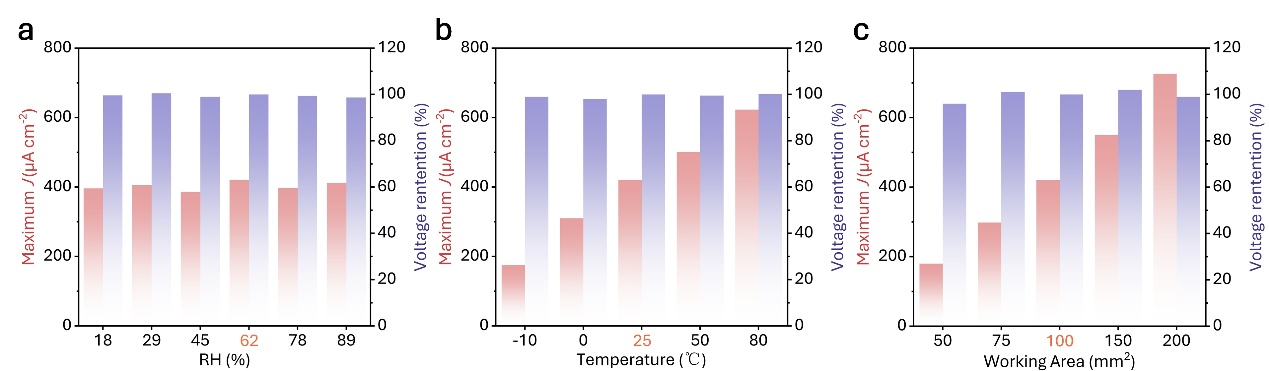


**Fig. S18 Impact of environmental factors on IOPS output power.** a and b, The impact of environmental factors on IOPS (the factors marked in red are the calculation reference values, the same below). Maximum current density and voltage holding rate under different relative humidity (RH%) and temperature. c, Maximum current density and voltage holding rate of IOPS under different working areas.


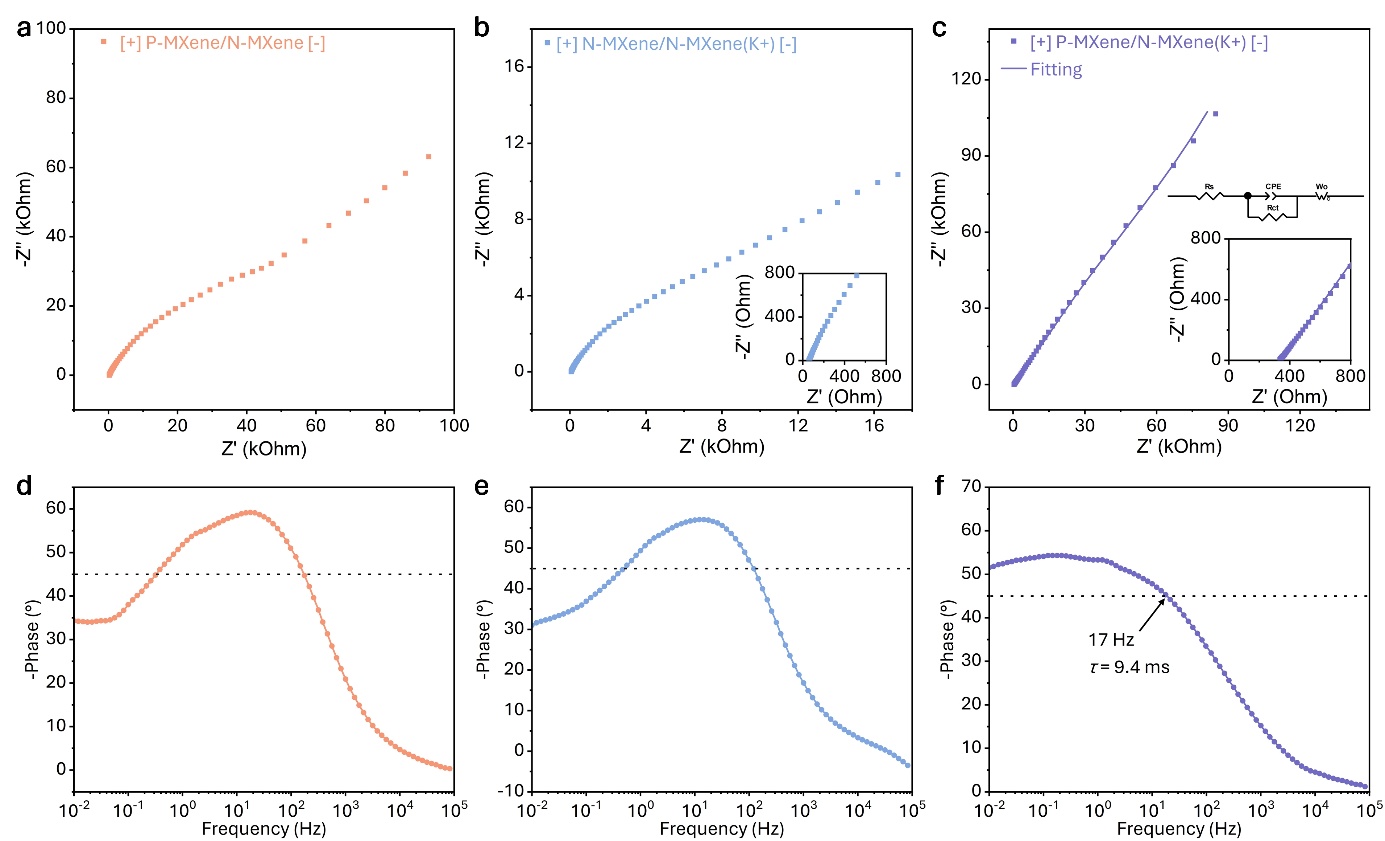


**Fig. S19 Electrochemical properties.** a-c, Complex plane representation of the real and imaginary parts of the impedance (Nyquist plots). Insets show magnified views. d-f, Impedance phase angle versus frequency (Bode angle plots).


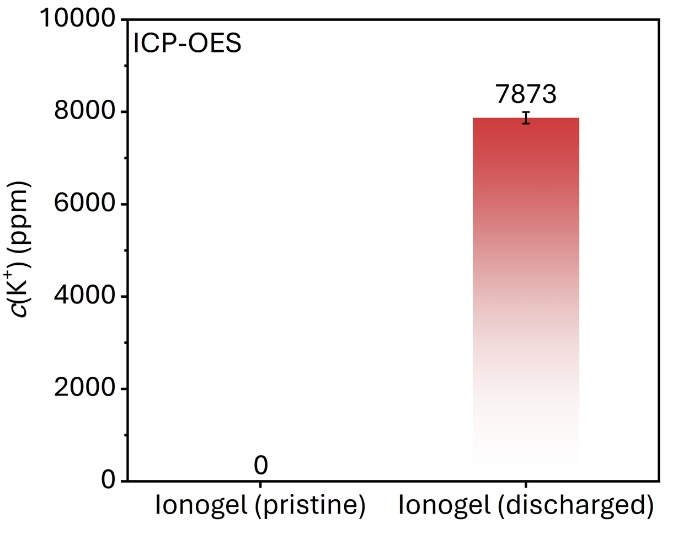


**Fig. S20 The elemental potassium concentration in the ion gel before and after discharge was measured using ICP-OES, indicating that K^+^ was transferred from N-MXene(K^+^) to the ionogel during discharge.** The data are presented as mean ±s.d., and the error bars represent the s.d. values measured from five independent devices.


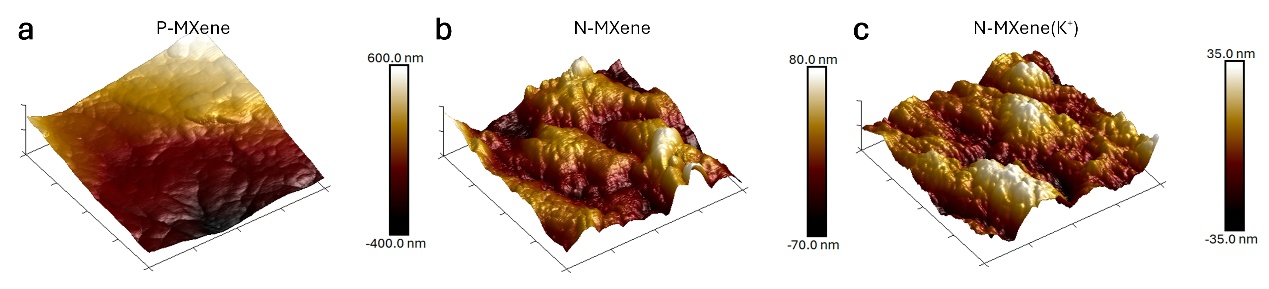


**Fig. S21 KPFM surface properties of N-MXene, N-MXene(K^+^), and P-MXene.**


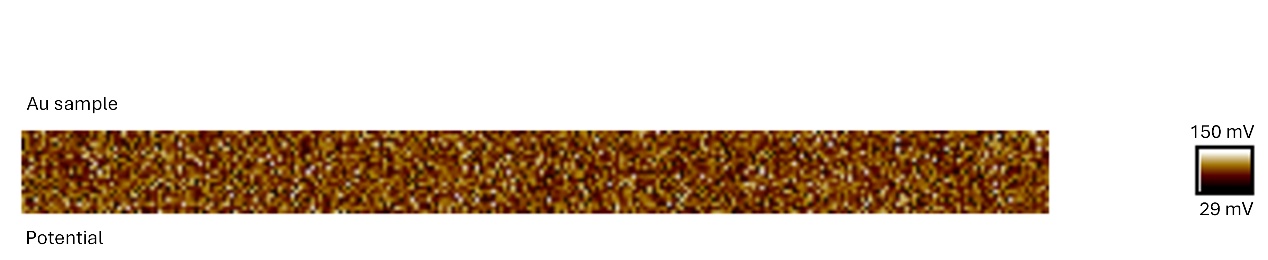


**Fig. S22 Contact potential difference of the gold standard sample.**


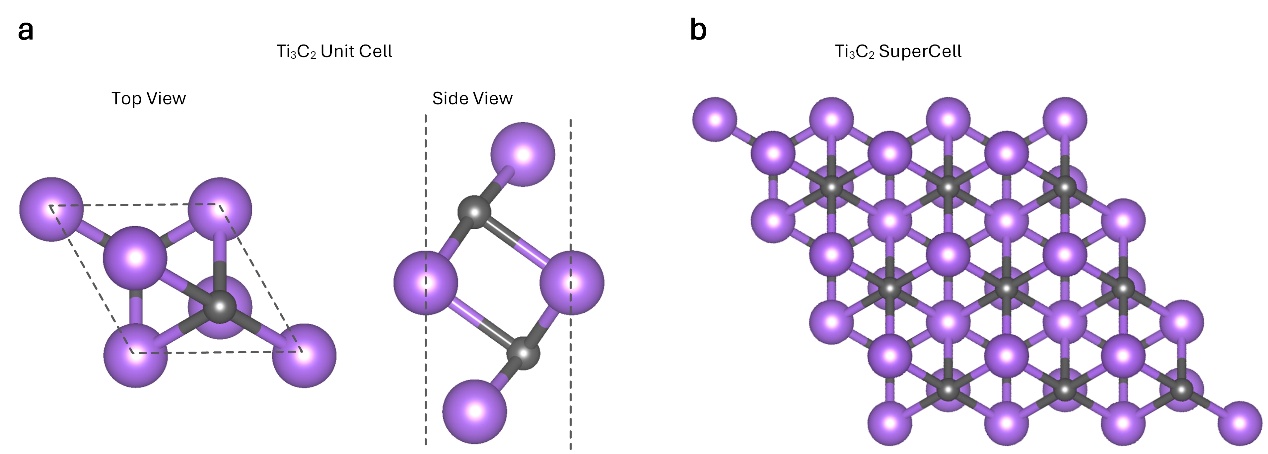


**Fig. S23 The unit cell model construction method of Ti_3_C_2_ MXene without the configuration of the capping group.** a, Top view and side view of the Ti_3_C_2_ unit cell model. b, Top view of the 3 x 3 supercell model of Ti_3_C_2_.


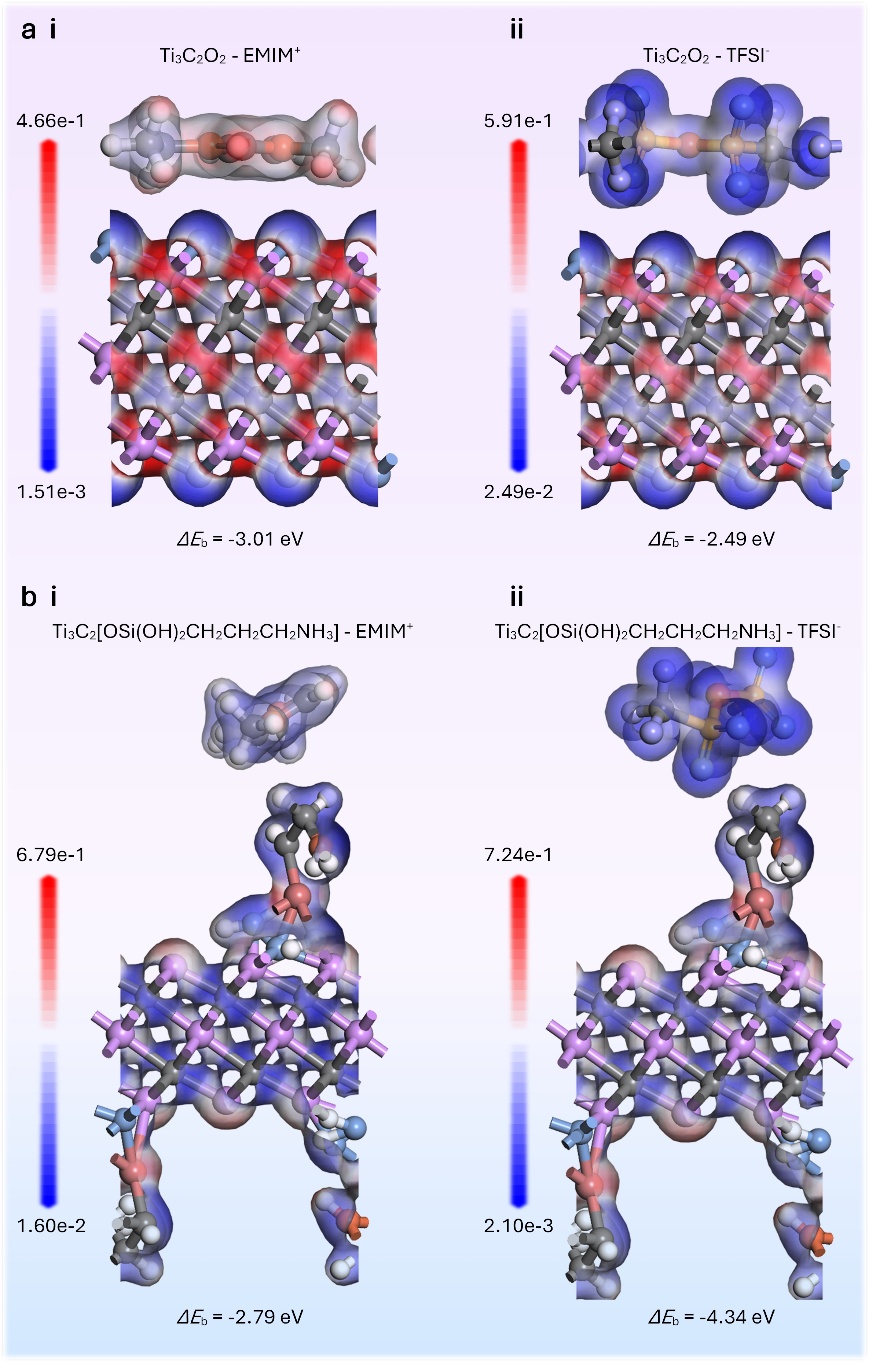


**Fig. S24 The interaction between positive and negative electrodes with ionic liquids.** a. The interaction and molecular electrostatic potential energy surface of N-MXene molecule (Ti_3_C_2_O_2_) with cation EMIM^+^ (i) and anion TFSI^-^ (ii). b. The interaction and molecular electrostatic potential energy surface of monodentate P-MXene molecule (Ti_3_C_2_[OSi(OH)_2_CH_2_CH_2_CH_2_NH_3_) with cation EMIM^+^ (i) and anion TFSI^-^ (ii). The scale unit is Hartree/e.


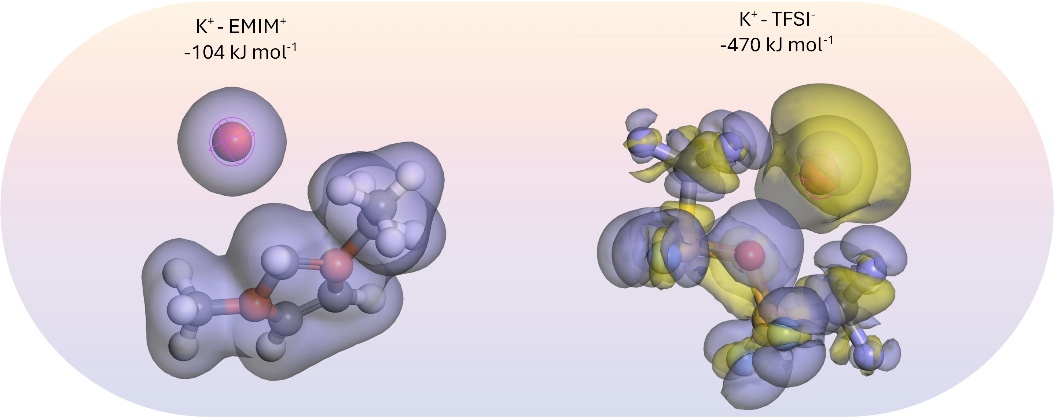


**Fig. S25 The affinity between K^+^ and ionic liquids is studied by combining deformation charge density and DFT calculation.** The figure shows the deformation charge density and calculated binding energy of K^+^-EMIM^+^ and K^+^-TFSI^-^.


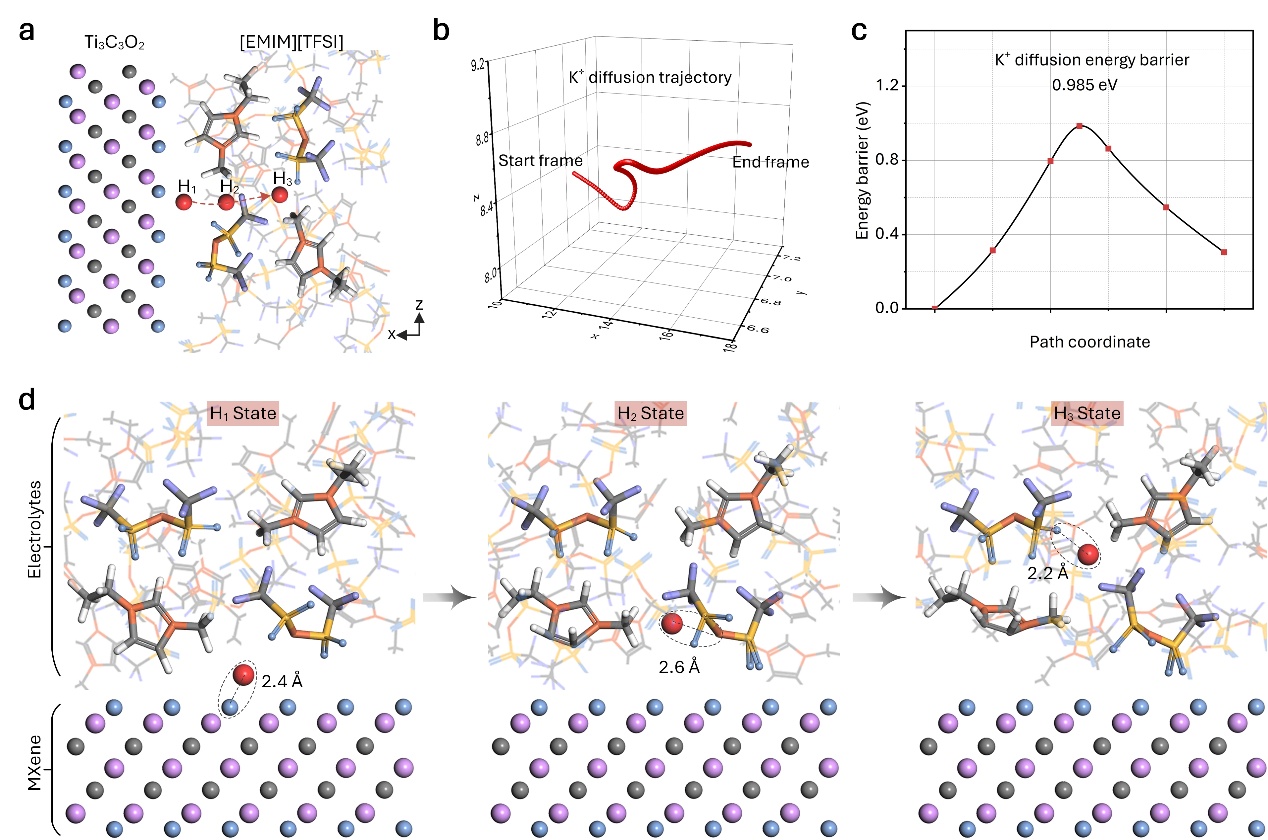


**Fig. S26 Screen shot of the K^+^ transport pathway at the IHP interface of N-MXene and ionic liquid.** a and b, K^+^ diffusion pathway. c, K^+^ migration barrier. d, K^+^ migration from H_1_ site to H_3_ site.

**S3 Supporting Tables**

**Table S1 | Related work on** **ion osmotic energy storage devices reported in the literature**

| Materials | Maximum output power density (μW cm^-2^) | Redox reaction involved | Cycle performance | Leakage and dry-up risks |
| --- | --- | --- | --- | --- |
| **Polarized MXene (This work)** | **17.5** | **None** | **50** | **None** |
| Bilayer of polyelectrolyte film [S10] | 5.52 | Involved | Water charging | Low |
| Gradient cellulose membrane [S11] | 4.9 | Involved | Water charging | High |
| Nanocellulose @ conductive MOF [S12] | 1500 | Involved | Water charging | High |
| Al_2_O_3_/PVDF [S13] | 18 | Involved | Water charging | High |
| Graphene oxide/Ionic liquids [S14] | 16.55 | None | Water charging | High |
| Stacked hydrogels [S15] | 2.7 | Involved | 10 | Medium |
| Hydrogel-Infused Paper [S16] | 180 | Involved | None | Medium |
| Ionic hydrogel [S17] | 12.43 | Involved | None | Medium |
| Living leaf [S18] | 0.014 | None | Water charging | High |
| Microbial biofilms [S19] | 1 | None | Water charging | High |

**Table S2 | Parameter setting of finite element simulation model**

| Parameter | Description | Value |
| --- | --- | --- |
| *A* | Length of ionogel | 150 μm |
| *B* | Width of ionogel | 100 μm |
| *C* | Width of MXene | 10 μm |
| *σ*_α_ | Space charge density of N-MXene(K^+^) | -6 C m^-2^ |
| *σ*_β_ | Space charge density of P-MXene | 4 C m^-2^ |
| *D*_K_  *D*_EMIM_  *D*_TFSI_  *ε* | Diffusion coefficient of K^+^  Diffusion coefficient of EMIM^+^  Diffusion coefficient of TFSI^-^  Dielectric constant | 1.96 × 10^−9^ m^2^ s^-1^  0.076 × 10^−9^ m^2^ s^-1^  0.076 × 10^−9^ m^2^ s^-1^  15 |

**Table S3 | Comparison of element content percentage**

| Element | Element content percentages | | |
| --- | --- | --- | --- |
|  | N-MXene | N-Mxene (K^+^) | P-MXene |
| Ti | 20.0 | 14.4 | 17.9 |
| C | 41.9 | 40.4 | 42.2 |
| O | 28.3 | 40.0 | 22.5 |
| F  K  N  Si | 9.88  0.00  0.00  0.00 | 4.58  0.62  0.00  0.00 | 7.64  0.00  3.58  6.29 |

**Table S4 | Sheet resistivity measured by four-probe technique**

| MXene Types | Sheet resistivity (mΩ cm) | | | |
| --- | --- | --- | --- | --- |
|  | 1 | 2 | 3 | Average |
| N-MXene | 96.5 | 94.3 | 102 | 97.6 |
| N-MXene(K^+^) | 142 | 156 | 144 | 147 |
| P-MXene | 3235 | 2951 | 3119 | 3101 |

**Table S5 | The open-circuit voltage of a folding-triggered IOPS with five units in series**

| Voltage recording | | | | | |
| --- | --- | --- | --- | --- | --- |
| Number of series | 1 | 2 | 3 | 4 | 5 |
| Voltage (V) | 0.56 | 1.12 | 1.69 | 2.27 | 2.82 |

**Supplementary References**

[S1] Y. Hu, H. Xiao, L. Fu, P. Liu, Y. Wu et al., Confined ionic-liquid-mediated cation diffusion through layered membranes for high-performance osmotic energy conversion. Adv. Mater. **35**(24), 2301285 (2023). https://doi.org/10.1002/adma.202301285

[S2] Y. Jia, F. Shi, H. Li, Z. Yan, J. Xu et al., Facile ionization of the nanochannels of lamellar membranes for stable ionic liquid immobilization and efficient CO_2_ separation. ACS Nano **16**(9), 14379–14389 (2022). https://doi.org/10.1021/acsnano.2c04670

[S3] Z. Yuan, B. Zhou, K. Yuan, Z. Xie, K. Zheng et al., High-aligned oppositely-charged nanocellulose/mxene aerogel membranes through synergy of directional freeze-casting and structural densification for osmotic-energy harvesting. Nano Energy. **124**, 109450 (2024). https://doi.org/10.1016/j.nanoen.2024.109450

[S4] L. Ding, M. Zheng, D. Xiao, Z. Zhao, J. Xue et al., Bioinspired Ti_3_C_2_T_x_ mxene-based ionic diode membrane for high-efficient osmotic energy conversion. Angew. Chem. Int. Ed. **61**(41), e202206152 (2022). https://doi.org/10.1002/anie.202206152

[S5] A. M. Sampaio, S. Bi, M. Salanne, L. J. A. Siqueira. Molecular dynamics simulations of ionic liquids confined into mxenes. Energy Storage Mater. **70**, 103502 (2024). https://doi.org/10.1016/j.ensm.2024.103502

[S6] A. M. Sampaio, J. F. R. V. Silveira, L. G. Dias, J. L. F. Da Silva, L. J. A. Siqueira. Adsorption of ionic liquids forming species on ti3c2tx mxenes surfaces by first-principle calculations. FlatChem. **35**, 100413 (2022). https://doi.org/10.1016/j.flatc.2022.100413

[S7] H. Sun. Compass:  an ab initio force-field optimized for condensed-phase applicationsoverview with details on alkane and benzene compounds. J. Phys. Chem. B **102**(38), 7338–7364 (1998). https://doi.org/10.1021/jp980939v

[S8] R. Akkermans, N. Spenley, S. Robertson. Compass iii: Automated fitting workflows and extension to ionic liquids. Mol. Simul. 1–12 (2020). https://doi.org/10.1080/08927022.2020.1808215

[S9] X. Wang, C. Tang, Q. Wang, X. Li, J. Hao. Selection of optimal polymerization degree and force field in the molecular dynamics simulation of insulating paper cellulose. Energies. **10**(9), 1377 (2017). https://doi.org/10.3390/en10091377

[S10] H. Wang, Y. Sun, T. He, Y. Huang, H. Cheng et al., Bilayer of polyelectrolyte films for spontaneous power generation in air up to an integrated 1,000 v output. Nat. Nanotechnol. **16**(7), 811–819 (2021). https://doi.org/10.1038/s41565-021-00903-6

[S11] S. Lee, J. Eun, S. Jeon. Facile fabrication of a highly efficient moisture-driven power generator using laser-induced graphitization under ambient conditions. Nano Energy **68**(104364 (2020). https://doi.org/10.1016/j.nanoen.2019.104364

[S12] S. Zhou, Z. Qiu, M. Strømme, C. Xu. Solar-driven ionic power generation via a film of nanocellulose @ conductive metal–organic framework. Energy Environ. Sci. **14**(2), 900–905 (2021). https://doi.org/10.1039/D0EE02730H

[S13] K. Ni, B. Xu, Z. Wang, Q. Ren, W. Gu et al., Ion-diode-like heterojunction for improving electricity generation from water droplets by capillary infiltration. Adv. Mater. **35**(40), 2305438 (2023). https://doi.org/10.1002/adma.202305438

[S14] Y. Han, Y. Wang, M. Wang, H. Dong, Y. Nie et al., Nanofluid-guided janus membrane for high-efficiency electricity generation from water evaporation. Adv. Mater. **36**(23), 2312209 (2024). https://doi.org/10.1002/adma.202312209

[S15] T. B. H. Schroeder, A. Guha, A. Lamoureux, G. VanRenterghem, D. Sept et al., An electric-eel-inspired soft power source from stacked hydrogels. Nature. **552**(7684), 214–218 (2017). https://doi.org/10.1038/nature24670

[S16] A. Guha, T. J. Kalkus, T. B. H. Schroeder, O. G. Willis, C. Rader et al., Powering electronic devices from salt gradients in aa-battery-sized stacks of hydrogel-infused paper. Adv. Mater. **33**(31), 2101757 (2021). https://doi.org/10.1002/adma.202101757

[S17] J. Fang, X. Zhang, P. Duan, Z. Jiang, X. Lu et al., Efficient and cold-tolerant moisture-enabled power generator combining ionic diode and ionic hydrogel. Mater. Horiz. **11**(5), 1261–1271 (2024). https://doi.org/10.1039/D3MH01496G

[S18] Q. Hu, X. Lin, G. Ren, J. Lü, W. Wang et al., Hydrovoltaic electricity generation induced by living leaf transpiration. Nat. Water. **2**(10), 988–998 (2024). https://doi.org/10.1038/s44221-024-00311-9

[S19] X. Liu, T. Ueki, H. Gao, T. L. Woodard, K. P. Nevin et al., Microbial biofilms for electricity generation from water evaporation and power to wearables. Nat. Commun. **13**(1), 4369 (2022). https://doi.org/10.1038/s41467-022-32105-6
